# Role of mitochondrial translation in modulating inflammatory disease outcome: Current knowledge and future perspectives

**DOI:** 10.1016/j.jbc.2026.111455

**Published:** 2026-04-17

**Authors:** Swarnali Basu, Rukshar Khan, Shiva Sharma, Priyanka Prajapati, Veena Ammanathan, Sumit Rungta, Amit Lahiri

**Affiliations:** 1 CSIR-Central Drug Research Institute, Department of Pharmacology, Lucknow, Uttar Pradesh, India; 2 Jawaharlal Nehru University, New Delhi, India; 3 Academy of Scientific and Innovative Research (AcSIR), Ghaziabad, India; 4 King George's Medical University, Department of Medical Gastroenterology, Lucknow, Uttar Pradesh, India

**Keywords:** mitochondria, oxidative phosphorylation, immune regulation, mito-nuclear communication, stress response pathways, inflammatory diseases, mitoribosomes (MRPS/MRPL), mitochondrial unfolded protein response (mtUPR), mitochondrial translation (MT)

## Abstract

Mitochondrial translation is crucial for maintaining cellular respiration, energy balance, calcium signaling, apoptosis, immune surveillance, and the regulation of inflammatory responses. This specialized process, involving mitochondrial rRNAs, tRNAs, mitoribosomes, and nuclear-encoded translation factors, ensures the synthesis of mitochondrially encoded proteins that support oxidative phosphorylation. The mitochondrial translation cycle is tightly regulated by RNA-binding proteins, mitochondrial unfolded protein response, and stress-responsive pathways such as mTOR, particularly during metabolic shifts and immune activation. Emerging evidence highlights mitochondrial translation as a critical modulator of inflammation. In this review, we describe alteration in mitochondrial-specific translation dynamics in immune cells, its adaptation to stress, and its interplay with organelle-wide signaling *via* mito-nuclear and mito-cytosolic communication. We focus on the alterations in mitochondrial translation machinery, including mitoribosomal proteins, rRNA, tRNA synthetases or other regulatory factors linked to inflammatory diseases, including neurodegeneration, IBD, metabolic and cardiovascular disorders. We further examine how mitochondrial translation influences immune responses through mitochondrial DNA/RNA release, activation of mitochondrial damage-associated molecular patterns, and inflammasomes such as NLRP3. Collectively, mitochondrial translation functions as an immune-centric checkpoint that presents a promising therapeutic target for intervention in inflammation-driven diseases.

Mitochondria are essential eukaryotic organelles that have a fundamental role in cellular bioenergetics, that produce producing over 90% of the cell's ATP through the TCA cycle and oxidative phosphorylation (OXPHOS) pathway (1). The TCA cycle functions as a central metabolic hub that generates high-energy electron carriers (NADH and FADH2), which directly fuel oxidative phosphorylation to drive ATP synthesis through the mitochondrial electron transport chain. Together, the TCA cycle and OXPHOS form a bioenergetic network coupling nutrient oxidation to efficient energy production and thereby maintain cellular homeostasis, redox balance and metabolic adaptability in both physiological and stress conditions. Mitochondria also actively participate in other cellular functions such as cell death, calcium homeostasis and regulation of immunity (Fig. 1).

**Figure 1:**
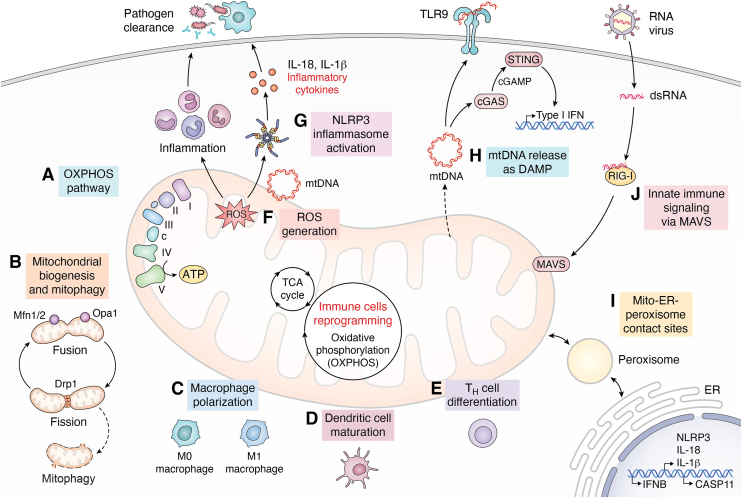
**Multifaceted role of mitochondria in innate and adaptive immune regulation.** This schematic illustrates the central role of mitochondria in orchestrating immune responses through dynamic signaling, metabolic adaptation, and inter-organelle communication. *A*, OXPHOS Pathway: Mitochondrial oxidative phosphorylation (OXPHOS) occurs in the inner mitochondrial membrane, where electron transfer through complex I-IV drive proton pumping to generate an electrochemical gradient. This proton motive force powers ATP synthase (complex V) to produce ATP, the primary energy currency of the cell. *B*, Mitochondrial biogenesis and Mitophagy: Mitochondrial biogenesis maintains mitochondrial network integrity and quality control through fission, fusion and mitophagy ensuring cellular energy homeostasis. Mitochondrial fusion mediated by Mfn1/2 and Opa1, promotes content mixing and maintenance of mtDNA integrity, whereas fission primarily driven by Drp1 facilitates mitochondrial segregation and turnover. Damaged mitochondria are removed through mitophagy, maintaining mitochondrial and immune homeostasis. *C*, Macrophage Polarization: Mitochondrial metabolism drives polarization of macrophages from pro-inflammatory M1 to anti-inflammatory M2 phenotypes. *D*, Dendritic Cell Maturation: Enhanced mitochondrial biogenesis and OXPHOS promote antigen presentation and maturation of dendritic cells. E, T Helper Cell Differentiation: Mitochondrial metabolic rewiring shapes the differentiation of Th1/Th2 cells during adaptive immune responses. *F*, Reactive Oxygen Species (ROS) Generation: Mitochondria generate ROS as a first-line response to stress and infection, promoting immune activation. G, NLRP3 Inflammasome Activation: ROS and mitochondrial damage lead to inflammasome assembly, resulting in the release of IL-1β and IL-18, thereby driving inflammation and pathogen clearance. *H*, mtDNA as a DAMP: Mitochondrial DNA released during stress serves as a damage-associated molecular pattern (DAMP), activating cGAS-STING signaling and Type I IFN responses, or engaging TLR9. *I*, Mito-ER-Peroxisome Contact Sites: Mitochondria form functional contacts with the endoplasmic reticulum and peroxisomes to coordinate immune signaling and inflammasome priming. *J*, Innate Immune Signaling *via* MAVS: RNA virus infection activates Rig-I-MAVS signaling at the mitochondrial surface, modulating downstream antiviral pathways.

Although more than 99% of the mitochondrial proteins are nuclear-encoded, the mitochondria contain its own genome (∼16.5 kb), which is present in multiple copies. Mitochondrial DNA (mtDNA) encodes 13 essential subunits of the respiratory chain complex that are present in the inner mitochondrial membrane (IMM) (2) along with two rRNAs (16s and 12s) and 22 tRNAs. The translation of the mitochondrial-encoded mRNAs occurs in the mitochondrial matrix, utilizing mitochondria-encoded tRNAs and nuclear-encoded mitochondrial ribosomal proteins (mitoribosomes) that are imported to mitochondria through the TOM-TIM translocase system (3). Defects in mitochondrial protein synthesis disrupt energy production, leading to mitochondrial dysfunction and a range of diseases with diverse clinical phenotypes (4). These pathologies are often linked to mutations or variations in nuclear or mitochondrial genes that regulate mitochondrial biogenesis (Fig. 2).

**Figure 2:**
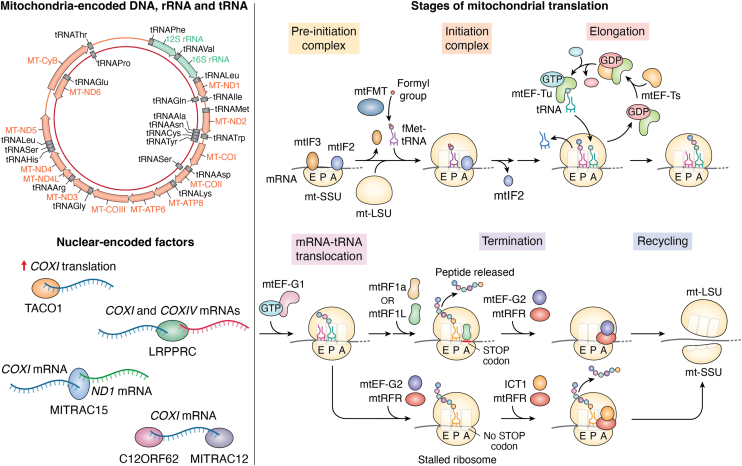
**Schematic representation of mitochondrial protein translation and its regulation.** Human mitochondrial genome (∼16.5 kb) is present in multiple copies and encodes 13 essential subunits of respiratory chain complex present in inner mitochondrial membrane (IMM), including Complex I (ND1, ND2, ND3, ND4, ND5, ND6, ND6L), Complex III (cytb), Complex IV (COXI, COXII, COXIII), and Complex V (ATP6 & ATP8). Additionally, mtDNA encodes two rRNAs (16s and 12s) and 22 tRNAs. There are also several nuclear-encoded factors that are important for mitochondrial translation. One of them is the Leucine-rich Penta-tri-co-peptide Repeat-containing Protein (LRPPRC), which stabilizes the transcripts of some genes and indirectly affects mitochondrial translation. Others like translational activator of cytochrome c oxidase I (TACO1), mitochondrial translation regulation assembly intermediate of cytochrome c oxidase 12 (MITRAC12), 15 (MITRAC15) and C12ORF62 (Cytochrome C Oxidase Assembly Factor COX14) indirectly or directly affect mitochondrial translation. Mitochondrial translation takes place in four steps: initiation, elongation, termination, and recycling. The process begins with the formation of the pre-initiation complex, comprising the small ribosomal subunit, mitochondrial initiation factors (mtIF3 and mtIF2), and mRNA. As translation progresses, mtIF3 dissociates, allowing the large ribosomal subunit to join, followed by the binding of formyl-Methionine-tRNA, mitochondrial formyl transferase (mtFMT) transfers the formyl group to methionine. The departure of mtIF2 marks the transition to the elongation phase, where mtEF-Tu delivers aminoacyl-tRNA to the A site through GTP hydrolysis, while mtEF-Ts facilitates GDP-to-GTP exchange to regenerate mtEF-Tu. Translocation of mRNA is catalyzed by mtEF-G1, advancing the ribosome along the transcript. Upon reaching a stop codon, mtRF1a/mtRF1L recognizes the termination signal and binds to the A site, releasing the synthesized peptide. Finally, mtEF-G2 and mtRFR promote ribosomal disassembly, leading to the release of both ribosomal subunits and mRNA, completing the translation cycle. In case of mRNA missing stop codon, C12ORF62 and ICT1 (Inducer of CBF1 Transcript 1) come into play and induce termination.

Mitochondrial biogenesis, mitophagy and dynamics are key processes that maintain mitochondrial quality and energy generation in the cell. Mitochondrial homeostasis comprises a balance between mitochondrial biogenesis *i.e.*. generation of new mitochondria, removal of damaged mitochondria by mitophagy and quality control mechanisms (fission, fusion, and mitochondrial unfolded protein response) to adapt under stress. To ensure mitochondrial quality control there is coordination between peroxisome proliferator-activated receptor-γ coactivator (PGC1-α), as the main regulator of mitochondrial biogenesis and PINK1-Parkin which results in the activation of protein synthesis required for mitophagy. Drp1, OPA1, Mfn1/2 are increased to undergo fission or fusion respectively to maintain mitochondrial morphology and function (Fig. 1).

Mitochondria are critical to process that direct immune surveillance in both innate as well as adaptive immunity and inflammatory signaling and the interplay between mitochondrial metabolism and immunity is tightly coupled, particularly in immune cells (T-cells, macrophages) (5). Mitochondrial dynamics and quality control are also essential for immune cell function (6, 7). However, under severe or chronic stress, immune cells such as macrophages, dendritic cells and T cells undergo a metabolic reprogramming of cells that involves shifting from mitochondrial oxidative phosphorylation to glycolysis. This reprogramming influences the immune cell phenotype from a pro-inflammatory state (M1-like) to an anti-inflammatory (M2-like) states, T_H_ cell differentiation, activation, and dendritic cell maturation (Fig. 1). During the changes in immune cell phenotype, there is inhibition of the mitochondrial translation which can directly initiate the mitochondrial stress response resulting in reduction in cytoplasmic translation significantly and an increase in critical signal pathways such as the Integrated stress response (ISR) and mitochondrial unfolded protein response (mtUPR). The ISR detects different forms of stress and reprograms global protein synthesis through eIF2α phosphorylation, linking environmental stress to adaptive gene expression. Furthermore, the mtUPR functions to produce mitochondrial chaperons and proteases, helping in the repair of the mitochondrial unfolded proteins (8).

Under stress conditions, including inflammation, mitochondrial dysfunction occurs, resulting in the production of mitochondrial-derived ROS (mtROS). Mitochondrial dysfunction also results in the release of mitochondrial DNA (mtDNA) and RNA (mtRNA), which act as danger-associated molecular patterns (DAMPs). These DAMPs are recognized by the Pattern recognition receptors (PRRs). These PRRs include RIG-I-like receptors (RLRs), Toll-like receptors (TLRs) or NOD-like receptors (NLRs) (Fig. 1) that trigger the immune response. This immune recognition enables mitochondria to function as a dynamic immune signaling platform (9).

This dynamic immune signaling pathways coordinate the cell’s response to infection, stress, and damage through interaction with other organelles like the ER and peroxisomes (Fig. 1). NLRP3 senses cellular stress, including mitochondrial damage or oxidative stress. NLRP3 assembles the inflammasome with ASC and caspase-1, and ultimately drives the release of IL-1β, IL-18, and pyroptotic cell contents.

MAVS activates antiviral signaling, leading to type I interferon production and NF-κB activation. On the other hand, the STING pathway detects cytosolic DNA *via* cGAS, activates STING and TBK1. This activation leads to IRF3 and NF-κB signaling, and results in the production of type I interferons and pro-inflammatory cytokines, leading to the clearance of the pathogens (Fig. 1).

Inflammation is a double-edged sword that serves as a protective mechanism against infection and tissue injury marked by cytokine production and immune cell infiltration for pathogen clearance but with well-settled resolution. Failure to resolve inflammation can result in chronic inflammatory diseases. Consequently, impairments in mitochondrial function can have widespread effects and are associated with numerous inflammatory human diseases (10). The synthesis of global mitochondrial proteins, along with the key signaling and regulatory proteins, supports the dual role of the mitochondria, both as the bioenergetics and immune cell metabolism. Therefore, investigating how mitochondrial translation contributes to inflammation and inflammation-related diseases can provide valuable insights into potential metabolic interventions for modulating inflammatory responses (10, 11, 12).

In this review, we summarize the key components and cycle of mitochondrial translation, highlighting unique features of initiation, the role of RBPs, and feedback systems that maintain translation homeostasis. We discuss how stress-induced mito-nuclear crosstalk activates pathways such as the mtUPR and ISR, generates stress-responsive RBPs and remodels mitoribosomes to restore cellular homeostasis. We provide an overview of how mitochondrial translation influences inflammation through mito-nuclear communication, mito-cytoplasmic signaling, and metabolic reprogramming, which together support immune cell activation, differentiation, and function. We further explore specialized translation in macrophages and dendritic cells, translational pausing, and its role in inflammation resolution. Finally, we outline how mitochondrial translation defects contribute to inflammatory diseases and how pharmacological modulation offers therapeutic potential.

## Mitochondrial translation component and cycle

Mitochondrial translation (MT) is the process by which mitochondrial mRNA (mt-mRNA) are translated by the mitoribosomes to synthesize proteins (13). The entire mitochondrial genome is transcribed from the mtDNA into long polycistronic transcripts, that undergo multiple processing steps to produce functional mRNAs. Although most mitochondrial proteins are nuclear-encoded, mtDNA encodes 13 polypeptides of complexes I, III, IV, and V, as well as 22 tRNAs and 2 rRNAs. Mitochondrial translation more closely resembles prokaryotic systems than the eukaryotic cytoplasm but features several unique adaptations from nuclear transcription and cytosolic translation (14). Mitochondrial translation uses a genetic code, specifically AGG and AGA function as stop codons and UGA codes for tryptophan. Mitochondrial translation also differ from cytosolic translation as mt-mRNA molecules lacks 5′- caps and poly(A) tails that are central for nuclear-encoded mRNAs (15, 16). Other differences are mitochondria translate all codons using just 22 tRNAs and a single tRNA^Met^ formylation by methionyl-tRNA formyltransferase signals that contribute to initiation processes by promoting large subunit joining (17) and elongation at the peptidyl transferase center of the mt-LSU(mitoribosomal large subunit) (18). The mt-SSU (mitoribosomal small subunit) binds mRNAs without requiring initiation factors or tRNA (19), initiating translation *via* mitochondrial-specific factors that recognize start codons directly (19, 20). MTRF1 (mitochondrial translation release factor) triggers translation termination, while MRRF (mitochondrial ribosome recycling factor) together with mtEFG2 (mitochondrial elongation factor) facilitate ribosome recycling and tRNA release (21) (Table 1).

**Table 1:** Key nuclear-encoded factors regulating mitochondrial translation stages

Factors	Function	Known mutations and diseases
Mitoribosome Regulation		
PTCD1	Regulates mitochondrial mRNA stability	Cardiomyopathy (128), Linked to Alzheimer’s Disease (129)
MRP family of proteins	Essential for Mitoribosome structure and function	MRP7- Congenital sensorineural deafness (130) MRP16 - Fatal neonatal lactic acidosis (131) MRP 22 - Cardiomyopathy, Tubulopathy, Hypotonia, Skin edema (132)
GTBP5	Late stage of mitoribosome large subunit assembly (133)	-
GTPBP10	Late stages of mt-LSU maturation (134)	-
Mt-tRNA processing		
RNase Z (ELAC2)	Cleaves pre-tRNAs to form mature tRNAs	Hypertrophic cardiomyopathy (135)
RNase P (Complexed with proteins MRPP1, MRPP2, MRPP3)	Cleaves the 5′ end of mt-tRNAs	Mitochondrial encephalomyopathy, lactic acidosis, Neurodevelopmental delay, cardiomyopathy (136)
tRNAs post-translational modifications		
TRMT10C & TRMT61B	Methyltransferases- to enhance tRNA stability	Hepatoblastoma Susceptibility (137), Aneuploid Cancers (138).
PUS1 & PUS3	For introducing pseudouridine modifications on mt-tRNAs.	Hepatocellular Carcinoma (139), Intellectual disability (140)

In the following sections, we outline the components and regulatory phases of mitochondrial translation and describe how this system adapts to cellular stress through mito-nuclear communication, mito-cytosolic signaling, RNA-binding proteins, and stress-driven metabolic reprogramming.

### Components of mitochondrial translation

#### Mitochondrial ribosomes (Mitoribosomes)

The nuclear-encoded mitoribosomes, primarily associated with the inner mitochondrial membrane, are specialized for synthesis of 13 mtDNA encoded OXPHOS proteins. The biogenesis of mitoribosomes occurs by a multi-step process that is tightly regulated. These ribosomes are composed of two subunits: the small 28S subunit (mt-SSU), consisting of 12S rRNA and 30 proteins, and the large 39S subunit (mt-LSU), comprising of 16S rRNA and 52 proteins. These subunits together assemble in the mitochondrial matrix to form functional 55S mitoribosomes (22). The 12S rRNA and 16S rRNA are transcribed from mtDNA and the majority of the ribosomal proteins (MRPs) are nuclear encoded. The MRPs are imported to the mitochondrial matrix through the TOM-TIM translocase system, where they are incorporated into a pre-ribosomal complex coordinated by chaperones, GTPases, helicases and RNA-modifying enzymes. For example, there are key proteins such as MRPL45, which anchors mitoribosomes to IMM (23) whereas Oxa1, positioned near the ribosomal exit tunnel, enables co-translational insertion of at least nine mitochondrial proteins (24). Mitoribosomes contain a higher ratio of proteins to rRNA as compared to their cytoplasmic counterparts. MRPs are often overproduced to compensate for slow mitoribosome assembly (25). Human mitoribosome assembly takes approximately 2 to 3 h, which is slower than the assembly of the bacterial ribosome. The slower assembly may be due to the nuclear genetic origin of MRPs, which are synthesized in excess, imported into mitochondria, and regulated by degradation of unassembled protein fractions. Once assembled, the functional mitoribosome engages with numerous nuclear-encoded factors to facilitate the distinct stages of protein synthesis. Defective mitoribosome subunits are targeted for degradation by activating the mitochondrial unfolded protein response (26). Thus, mitoribosome regulation ensures controlled mitochondrial translation supporting efficient energy production and hence maintain cellular homeostasis.

#### Nuclear-encoded translation factors

Mitochondrial DNA (mtDNA) transcription is entirely dependent on nuclear-encoded factors. Transcription is initiated by POLRMT (mitochondrial RNA polymerase), while TEFM (transcription elongation factor) enhances transcript elongation, resulting in the production of genome-length polycistronic transcripts (27). The three primary polycistronic transcripts produced are processed into mature RNAs (tRNA, rRNA, mRNA) within specialized mitochondrial RNA granules. RNA maturation follows the ‘tRNA punctuation’ model, where interspersed tRNAs are excised to release rRNAs and mRNAs (28). This process is driven by nuclear-encoded enzymes such as mitochondrial RNase P (Ribonuclease P) and RNase Z (Ribonuclease Z/ELAC2) that cleave the 5′ and the 3′ ends of tRNAs, thereby releasing individual mt-tRNAs, mt-mRNAs, and mt-rRNAs. However, certain mRNAs lack flanking tRNAs and are thus processed *via* non-canonical maturation pathways (29).

#### Mitochondrial-encoded DNA, rRNA and tRNA

This section will begin with a highlight of the key components of the mitochondrial translation that are encoded in the organelle. Furthermore, there is a discussion of key base modifications and the processes that are critical for the functions of these RNAs.

• mtDNA: Human mitochondrial genome (∼16.5 kb) is present in multiple copies and encodes 13 essential subunits of respiratory chain complex present in inner mitochondrial membrane (IMM), including Complex I (ND1, ND2, ND3, ND4, ND5, ND6, ND6L), Complex III (cytb), Complex IV (COXI, COXII, COXIII), and Complex V (ATP6 & ATP8). Additionally, mtDNA encodes two rRNAs (16s and 12s) and 22 tRNAs.
• rRNAs: Mitochondrial rRNAs (12S and 16S) and tRNAs are transcribed from the polycistronic heavy strand of mtDNA and require precise processing to become functional. The mature 12S and 16S rRNAs form the structural core of the 28S and 39S mito-ribosomal subunits, respectively, with the large subunit incorporating either tRNA^Phe^ or tRNA^Val^. Assembly and stabilization of mitoribosomes are coordinated by several nuclear-encoded factors, including chaperones, GTPases, ATP-dependent helicases, and RNA-modifying enzymes (30). Unlike cytoplasmic rRNA, mitochondrial rRNA is transcribed, processed and assembled inside the mitochondria. Although mitochondrial rRNAs undergo minimal chemical modification, their integration with nuclear-encoded ribosomal proteins is tightly regulated. After folding and chemical modification (methylation, pseudouridylation), they combine with imported nuclear-encoded ribosomal proteins in the mitochondrial matrix to assemble the small and large mito-ribosomal subunits.
• tRNAs: Mitochondria encode 22 tRNAs, all transcribed by mtDNA and depend on nuclear-encoded aminoacyl-tRNA synthetases for charging. These mt-tRNAs also undergo post-transcriptional modifications to ensure proper folding and translation efficiency. Similarly, mt-rRNAs (12S rRNA and 16S rRNA) provide the structural framework for the ribosome and are also post-translationally modified to promote the fidelity of mitochondrial translation. The ability to translate 13 polypeptides with limited tRNAs is made possible by relaxed codon-anticodon pairing rules and non-standard base pairing (wobble base pairing), allowing a single tRNA to recognize multiple codons (Fig. 2).

Mt-tRNAs undergo a distinct set of post-transcriptional modifications essential for their stability, folding, and accurate decoding like methylation (*e.g.*, m^1^A, m^5^C), thiolation (31), and mitochondria-specific modifications like taurinomethylation (τm^5^U and τm^5^s^2^U) at the wobble base (U34) which are unique and crucial for codon recognition. Additionally, pseudouridylation by PUS1 and PUS3 contributes to tRNA structural integrity and translation fidelity (31). Depending on the affected tRNA or modifying defect, these genetic lesions can lead to neurodevelopmental delay, hepatocellular carcinoma, and intellectual disability, depending on the tRNA and modifying enzyme involved (Table 1). Mitochondrial aminoacyl-tRNA synthetases (mt-aaRSs) charge mitochondrial tRNAs with their respective amino acids. There are 19 mt-aaRSs, each specific to a standard amino acid, except for glutamine, which lacks a dedicated synthetase in mitochondria. Instead, mitochondrial tRNA^Glu^ synthetase indirectly charges tRNA^Gln^. Mitochondrial GluRSs discriminates tRNA^Gln^ from tRNA^Glu^ through specific identity elements located in the anticodon and acceptor stem. The UUG anticodon and characteristic acceptor-stem base-pair signatures of tRNA^Gln^ function as recognition determinants. These features are selectively recognized by GluRS, allowing the enzyme to bind and aminoacylate only tRNA^Gln^.

Mutations in mt-tRNA genes can affect tRNA folding, stability, or modifications, leading to defective mitochondrial translation. Point mutations such as m.3243A>G in tRNA^Leu^ (UUR) (causing MELAS mitochondrial encephalopathy, stroke-like episodes, and lactic acidosis) and m.8344A>G in tRNA^Lys^ (32, 33) (causing MERRF myoclonic epilepsy with ragged red fibers) impairs translation and OXPHOS. The nature of these mutations includes point mutations, deletions, or nucleotide substitutions affecting the anticodon loop, acceptor stem, or regions involved in post-transcriptional modifications critical for tRNA function. For a comprehensive overview of all mitochondrial aminoacyl-tRNA synthetases (mt-aaRSs) implicated in disease, we encourage readers to refer to Tables 1 and 2. Mutations and diseases in other key nuclear-encoded factors that regulate different stages of mitochondrial translation are also mentioned in Table 1.

**Table 2:** Factors involved in translation initiation, elongation, termination and recycling

Mitotranslation initiation		
MTIF2	Promotion of the binding of the initiator tRNA to the small subunit of the ribosome.	Pathological Myocardial Hypertrophy (10)
MTIF3	Promotes the formation of the initiation complex on mitochondrial 55S ribosomes	Parkinson’s Disease (141)
TACO1	Mitigation of polyproline-induced stalling of the human mitochondrial ribosome (61)	Late-onset Leigh Syndrome (42)
Mitotranslation elongation		
EF-Tu	Delivers aminoacyl-tRNAs to the A site of the mitochondrial ribosome.	Lactic acidosis and Fatal Encephalopathy (10)
EF-Ts	nucleotide exchange factor	Early-onset encephalopathy (10)
EF-G1	tRNA translocation	Early-onset Leigh Syndrome (10)
Mitotranslation Termination and Recycling		
MTRF1L	Termination of translation in mitochondria, specifically at non-canonical stop codons	Loss of Cell Viability (142)
MTG1	Mitochondrial ribosome assembly and translational Activity	Pathological Cardiomyopathy (133)
Translation factors		
TFAM	Maintenance, expression and of mitochondrial DNA	Neurodegenerative Diseases (143)
PGC-1α	Mitochondria Biogenesis	Alzheimer’s Disease (144)
Mitochondrial Proteases		
LONP1	Proteostasis, degradation of unfolded and oxidatively damaged Proteins	Cerebral, ocular, dental, auricular and skeletal anomalies (CODAS) Syndrome (145)
ClpXP	Proteostasis	Infertility and sensorineural hearing loss (146)

Some of the key nuclear-encoded factors that regulate different stages of mitochondrial translation, such as mitoribosome regulation (GTBPs, MRP family of proteins), tRNAs processing and its post-translational modifications (TRMTs, PUS), mitochondrial proteases (LONP1 and ClpXP) and several other factors involved in translation initiation, elongation, termination and recycling are given in Table 2.

### Major steps in the mitochondrial translation cycle

There are four major steps in mitochondrial translation: initiation, elongation, termination, and ribosome recycling, along with the regulatory mechanisms that fine-tune this process in response to cellular and metabolic cues. A brief overview of the mitochondrial translation process is detailed in Figure 2.

#### Initiation

Mitochondrial translation initiation is a highly coordinated process that begins with mtIF3 binding to the 28S small subunit of the mitoribosome, ensuring proper subunit dissociation and creating a competent platform for mRNA loading. Subsequently, mtIF2, in its GTP-bound form, recruits the formylated initiator tRNA (mt-tRNA^fMet^) generated by the enzyme MTFMT to the P-site, where the mitochondrial mRNA aligns directly with the start codon. Then the 39S large subunit association completes the formation of the 55S initiation complex, and GTP hydrolysis by mtIF2 triggers the release of initiation factors, allowing elongation to proceed. Mutations in any of the initiation factors disrupt this initiation machinery, leading to impaired mitochondrial protein synthesis and resulting in diseases such as encephalomyopathy, leukoencephalopathy, cardiomyopathy, and severe neonatal mitochondrial disease (Fig. 2).

#### Elongation

Following initiation, the A-site of the 55S mitoribosome accepts aminoacylated tRNAs delivered by EF-Tu homolog TUFM, which forms a ternary complex with GTP and aa-tRNA. Correct codon-anticodon pairing triggers GTP hydrolysis, mediated by TUFM, allowing tRNA accommodation into the A-site. The peptidyl transferase centre of the 39S large subunit catalyzes peptide bond formation, transferring the nascent chain from the P site tRNA to the A site tRNA. Subsequently, EF-G1mt (GFM1) promotes ribosomal translocation, shifting the mRNA-tRNA complex by one codon, while EF-Ts homolog TSFM regenerates TUFM-GTP for the next cycle. This coordinated elongation cycle ensures accurate and efficient mitochondrial protein synthesis (Fig. 2).

#### Termination

Mitochondrial translation termination occurs when a stop codon - UAA or UGA - enters the A-site of the 55S mitoribosome, signaling the end of polypeptide elongation. This step is mediated primarily by the release factor mtRF1a (MTRF1L), which recognizes the stop codon and catalyzes hydrolysis of the ester bond linking the completed polypeptide to the P-site tRNA. In cases of stalled ribosomes or incomplete termination, factors such as ICT1 (MRPL58) and C12orf65(MTRFR) rescue peptidyl-tRNA species, promoting ribosome recycling and maintaining translational fidelity. Following peptide release, the ribosome recycling factor (RRF1, MRRF) and the GTPase EF-G2mt (GFM2) mediate subunit dissociation and preparation for a new initiation cycle. Fibroblasts from C12orf65 (MTRFR)-mutated patients exhibit a global and consistent decrease in mitochondrial translation, resulting in defective OXPHOS assembly (34) (Fig. 2). Moreover, mutations in C12orf65 (MTRFR) have also been reported to cause optic atrophy, ophthalmoplegia, and Leigh syndrome (35).

#### Recycling

Mitochondrial ribosome recycling is the final phase of translation, ensuring that post-termination complexes are efficiently disassembled and made available for subsequent rounds of protein synthesis. After peptide release, the mitoribosome remains bound to mRNA and deacylated tRNA, forming a post-termination complex that must be resolved. This process is driven by the ribosome recycling factor (RRF1/MRRF) and the GTPase EF-G2mt (GFM2), which together promote the dissociation of the 55S mitoribosome into its 39S and 28S subunits, release of mRNA and tRNA, and clearance of residual translational components. The activity of mtIF3 then stabilizes the separated small subunit, preventing premature reassembly (Fig. 2).

### Mechanisms of mitochondrial Translation regulation

Mitochondrial translation is tightly regulated by processes not yet understood process essential for maintaining respiratory chain function. Core mechanisms controlling mitochondrial protein synthesis include translation initiation, RNA-binding proteins, and coordinated co-translational assembly of nuclear- and mitochondrial-encoded respiratory chain subunits (36).

Mitochondria translation initiation is distinct from the cytosolic translation as mt-mRNAs lack a significant length of 5′-UTR, with sequences only a few nucleotides preceding the start codon. Initiation factors such as mtIF2 and mtIF3 play crucial roles in forming two distinct pre-initiation steps, essential for proper initiation complex assembly. Recent studies have demonstrated that MTIF3 is critical for adipocyte mitochondrial function, with its depletion leading to impaired OXPHOS complex assembly, reduced respiration, and altered expression of mtDNA-encoded genes (36, 37).

Maintaining protein homeostasis in mitochondria is further complicated by the non-stoichiometric synthesis of multimeric respiratory complex subunits, with synthesis rates varying up to 32-fold between subunits (38). Despite this, cells achieve coordinated production across nuclear and mitochondrial genomes, likely to avoid proteotoxic stress from unassembled proteins (39). Translation regulation also involves RNA-binding proteins such as LRPPRC, which influences the stability and translation of COX1 and COX3 mRNAs (40) and may coordinate mitochondrial and nuclear gene expression (41). Similarly, TACO1 binds to mt-*CO1* mRNA and acts as an activator of *COX1* translation, along with the help of mitoribosomes. Mutation in TACO1 reduces mitochondrial translation, leading to diseases such as cardiac hypertrophy, motor dysfunction and late-onset visual impairment (42).

In mammalian mitochondria, feedback regulation ensures mitochondrial translation adapts to the influx of nuclear-encoded OXPHOS subunits. Factors involved in the early assembly of these subunits include C12orf62 and MITRAC12, which regulate COX1 (Complex IV) synthesis, while MITRAC15 influences both COX1 and ND2 (Complex IV and I) (43, 44, 45). In the absence of nuclear-encoded subunits, mitochondrial translation stalls at early assembly stages, resuming only when these subunits arrive to complete polypeptide synthesis and OXPHOS complex assembly.

A further regulatory layer operates through feedback systems that couple translation to respiratory chain assembly. Early assembly factors such as C12orf62, MITRAC12, and MITRAC15 monitor the availability of nuclear-encoded partner subunits and adjust the synthesis of mtDNA-encoded components accordingly. When nuclear-encoded assembly partners are insufficient, mitochondrial translation pauses at defined checkpoints, preventing the production of incomplete subunits that could disrupt mitochondrial proteostasis. Once the necessary components become available, translation resumes, ensuring coordinated and efficient assembly of OXPHOS complexes. Through these interconnected mechanisms, mitochondrial translation remains responsive yet tightly controlled under homeostatic conditions (Table 3).

**Table 3:** Nuclear-encoded factors facilitating ribosome maturation

Functional Class	Protein/Complex	Function	Importance
Mitoribosome-Associated RBPs	MRPS24, MRPS27, MRPL1, MRPL12	Structural components of mitoribosomes; stabilize and bind mt-rRNA	Essential for ribosome assembly and function
	C4orf14, C6orf203	Ribosomal assembly factors	Nuclear-encoded, imported for ribosome maturation
	RBFA	Facilitates 12S rRNA maturation and small subunit assembly	rRNA-binding assembly chaperone
	PTCD3	Interacts with 12S rRNA and mitoribosomes	Supports ribosome function and translation elongation
Translational Regulators (RBP Function)	TACO1	Specific translational activator for MT-CO1 mRNA	Deficiency linked to Leigh syndrome
	GRSF1	Binds G-rich regions in mtRNA; promotes processing and translation	Also localizes to RNA granules
	LRPPRC-SLIRP Complex	Stabilizes polyadenylated mt-mRNAs and supports translation	Mutation causes French-Canadian Leigh syndrome
	MTPAP (formerly PAPD1)	Adds poly(A) tails to mt-mRNAs; enhances stability	Nuclear-encoded poly(A) polymerase
mtRNA Processing and Localization RBPs	C1QBP	Functions as an RNA chaperone; supports mtRNA folding and processing	Less well-characterized in translation initiation
	FASTKD2, FASTKD3, FASTKD5	Process and stabilize mitochondrial precursor RNAs	FASTKD5 also localizes to RNA granules
	PTCD1, PTCD2	Regulate mitochondrial tRNA and RNA processing	Involved in transcript maturation
tRNA Modification Proteins (RNA-Associated)	TRMU	tRNA 2′-O-methyltransferase; stabilizes mitochondrial tRNAs	Binds tRNA, not an RBP in the classical sense
Translation Termination & Quality Control Factors	MTRF1L	Recognizes UAA/UAG codons and releases nascent peptides	Not classically called an RBP but ribosome-associated
	ICT1	Rescue factor that releases stalled ribosomes	Integrated into mitoribosome large subunit

## Stress-responsive regulation of mitochondrial translation

Mitochondria dynamically regulate their translation machinery to sustain cellular homeostasis under physiological and pathological stress conditions. This adaptation is tightly integrated with broader cellular signaling networks and inflammatory responses. Given their dual genetic origin, mitochondrial translation is modulated by mitonuclear communication, protein homeostasis mechanisms, RNA-binding proteins, and metabolic sensors (46). Dysregulation of these regulatory layers not only impairs mitochondrial function but also activates inflammatory cascades, positioning mitochondrial translation at the crossroads of stress adaptation and immune signaling.

Mitochondrial translation is a finely tuned process that ensures the accurate synthesis of mtDNA-encoded respiratory chain proteins, enabling cells to maintain efficient oxidative phosphorylation under normal physiological conditions. Although the mitochondrial genome encodes only a small fraction of the proteins required for respiration, the fidelity and regulation of their synthesis are essential for preserving OXPHOS integrity. One of the most distinguishing features of mitochondrial translation is its unique mode of initiation. As noted above, mitochondrial mRNAs lack 5′ UTRs, they rely on specialized mechanisms for start codon recognition. Initiation factor such as mtIF3 initially helps maintain the small mitoribosomal subunit in a specific conformation and likely prevent premature binding of the large subunit or incorrect mRNA/tRNA binding, thus ensuring fidelity of initiation, whereas mtIF2 then facilitates the binding of the correct initiator tRNA (fMet-tRNA) to the small subunit, stabilizing this complex. Recent studies showed that MTIF3 depletion in adipocytes leads to defective OXPHOS assembly, reduced respiration, and disrupted expression of mtDNA-encoded genes, demonstrating how sensitive mitochondrial function is to alterations at the earliest stages of translation (37).

**Figure 3:**
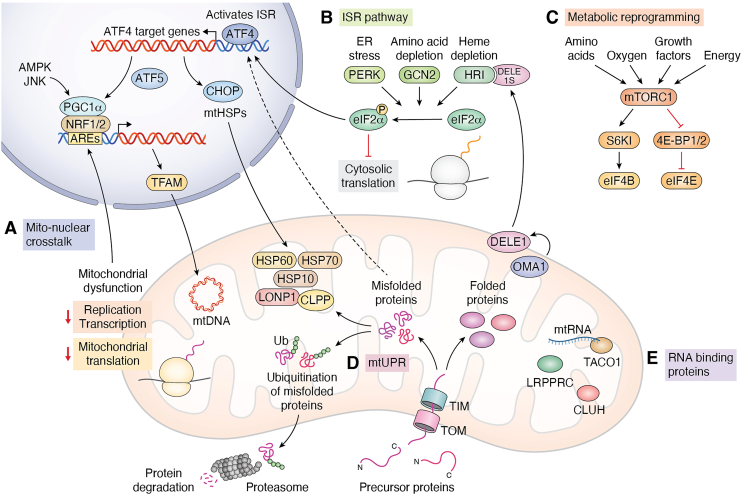
**Integrated Stress-Responsive Regulation of Mitochondrial Translation and Cellular Homeostas**is. *A*, Mito-Nuclear Crosstalk: Mitochondrial dysfunction marked by translation defects, oxidative stress, and ATP depletion activates retrograde signaling to the nucleus through ATF4. This triggers the transcription of key nuclear regulators (including NRF1, NRF2, POLRMT, TFAM) facilitating mitochondrial biogenesis and recovery. Transcriptional coactivators PGC-1α amplify this response. *B*, Integrated Stress Response (ISR): Stress sensors (PERK, GCN2, HRI) phosphorylate eIF2α, initiating the Integrated Stress Response (ISR), which globally attenuates translation while promoting selective synthesis of stress regulators like ATF4 and CHOP. The DELE1-OMA1-HRI axis further links mitochondrial dysfunction to cytosolic ISR signaling. *C*, Metabolic Reprogramming: In immune and proliferating cells, mitochondrial metabolism adapts by shifting between glycolysis and OXPHOS. These shifts are mediated by changes in mitochondrial translation that adjusts the TCA cycle and respiratory chain activity. Mitochondrial transcription and translation are upregulated in response to cellular energy demands, supporting immune cell functions and tissue homeostasis. mTORC1, sensing amino acids, oxygen, and energy status, regulates translation through effectors like S6K1, 4E-BP1/2, and eIF4B, thereby integrating growth signals with mitochondrial function. This axis governs anabolic responses and mitochondrial translation during growth, cellular stress and immune activation. *D*, Mitochondrial unfolded protein response (mtUPR): The mitochondrial unfolded protein response (mtUPR) is activated upon accumulation of misfolded proteins in the mitochondrial matrix. Proteases such as LONP1 and CLPP, along with chaperones HSP60, HSP70, and HSP10, maintain mitochondrial proteostasis by promoting correct protein folding and degrading damaged proteins. This ensures the integrity of respiratory chain components and modulates cytosolic translation. Under mitochondrial stress, defective import of nuclear-encoded mitochondrial proteins leads to their ubiquitination and proteasomal degradation, safeguarding cytosolic protein homeostasis and preventing proteotoxicity. *E*, RNA-binding proteins: RNA-binding proteins (RBPs) such as LRPPRC, TACO1, and CLUH play critical roles in post-transcriptional regulation of mtDNA. LRPPRC stabilizes mitochondrial mRNAs and promotes their polyadenylation and translation, whereas TACO is a mitochondrial translational activator specifically required for efficient translation of mitochondrial encoded COX1 (cytochrome c oxidase subunit I). CLUH binds to nuclear-encoded mitochondrial mRNAs in the cytoplasm and regulates their stability, localization, and translation, contributing to proper mitochondrial biogenesis.

When cells encounter metabolic, inflammatory, or environmental stress, the regulatory pathways that maintain mitochondrial translation under homeostasis are rapidly reconfigured to protect mitochondrial integrity and restore cellular homeostasis. Mitochondria communicate their distress to the nucleus through retrograde signaling pathways, while the nucleus adjusts mitochondrial transcription and biogenesis through anterograde signals. This bidirectional communication allows mitochondrial translation to adapt dynamically to fluctuations in energy demand, oxidative stress, or proteotoxic pressure. This adaptation is tightly integrated with broader cellular signaling networks and inflammatory responses. Given their dual genetic origin, mitochondrial translation is modulated by mito-nuclear communication, protein homeostasis mechanisms, RNA-binding proteins, and metabolic sensors (46). Dysregulation of these regulatory layers not only impairs mitochondrial function but also activates inflammatory cascades, positioning mitochondrial translation at the crossroads of stress adaptation and immune signaling. In this section, we will discuss about the mechanisms by which mitochondrial translation is regulated under homeostatic and stress conditions in detail (Fig. 3).

### Mito-nuclear communication in response to mitochondrial stress

The nucleus maintains mitochondrial biogenesis through a hierarchy of transcription factors and co-activators that regulate both nuclear-encoded mitochondrial proteins and mitochondrial DNA expression. At the core of this control system are NRF1 and NRF2, two transcription factors that activate the expression of a large number of nuclear genes required for mitochondrial function (47). A crucial downstream target of NRF1 and NRF2 is TFAM (mitochondrial transcription factor A), the principal regulator of mtDNA packaging, transcription, and replication. When TFAM expression increases, mitochondrial DNA becomes more transcriptionally active, leading to elevated production of the 13 mtDNA-encoded respiratory chain subunits as well as the rRNAs and tRNAs required for mito-ribosome function. POLRMT, the mitochondrial RNA polymerase, is another key nuclear-encoded factor whose expression and activity dictate the rate of mtDNA transcription (48). Through the coordinated control of TFAM, POLRMT, and other mitochondrial transcription factors, the nucleus essentially determines how much mtDNA is transcribed and how efficiently mitochondrial translation can proceed (Fig. 3).

At a higher regulatory level, mitochondrial biogenesis is tightly coupled to the cell’s metabolic and energetic status through PGC-1α, a master co-activator that integrates environmental and physiological cues. PGC-1α does not bind to DNA directly but interacts with NRF1, NRF2, and other transcription factors to amplify their transcriptional output. When cellular energy levels fall, NAD^+^ increases, activating the deacetylase SIRT1, which in turn deacetylates and activates PGC-1α. Similarly, Calcium/Calmodulin-Dependent Protein Kinase IV (CaMKIV), activated by intracellular calcium fluctuations, phosphorylates PGC-1α to enhance its activity (49). Through these modifications, PGC-1α acts as a metabolic sensor that translates changes in nutrient availability, stress, or muscle contraction into increased transcription of nuclear genes encoding mitochondrial components (50). As a result, when cellular energy demand rises, PGC-1α drives a coordinated upregulation of nuclear-encoded OXPHOS subunits, mitochondrial transcription and replication factors, and key proteins involved in mitochondrial protein import, fatty acid oxidation, and antioxidant defence (Fig. 3).

Under stress (*e.g.* mitochondrial dysfunction or oxidative stress), mitonuclear communication enables a coordinated adjustment of both mitochondrial and nuclear gene expression. In the anterograde direction, nuclear regulators such as NRF1, NRF2, TFAM, TFB2M, and POLRMT enhance mtDNA transcription and replication by increasing the expression of proteins responsible for mtDNA packaging, promoter recognition, and RNA synthesis. As TFAM and POLRMT levels rise, mtDNA becomes more accessible and transcriptionally active, allowing mitochondria to boost the production of respiratory chain subunits and RNA components needed for translation. The coactivator PGC-1α amplifies this response by sensing metabolic stress through NAD^+^/SIRT1-mediated deacetylation or CaMKIV-dependent phosphorylation, which increase its activity. Once activated, PGC-1α turns on a wide set of genes that help cells make more mitochondria, use nutrients more efficiently, and adapt their metabolism to changing needs.

At the same time, mitochondria initiates retrograde signaling to inform the nucleus of impaired function. Accumulation of ROS, declining ATP levels, or stalled mitochondrial translation activate stress-sensitive kinases such as AMPK, JNK, and NRF2. AMPK detects shifts in the cellular AMP/ATP ratio and promotes transcriptional programs that restore energy balance by enhancing catabolism and limiting energy-consuming processes. JNK responds to oxidative or metabolic stress and activates nuclear genes involved in repair, inflammation control, and apoptosis prevention. Meanwhile, oxidative stress stabilizes NRF2 by blocking its degradation; NRF2 then translocate to the nucleus, where it induces antioxidant enzymes and detoxification pathways that help mitigate mitochondrial ROS. Through these combined anterograde and retrograde mechanisms, cells continually adjust mitochondrial translation to restore homeostasis and support survival during stress (Fig. 3).

Importantly, this bidirectional communication suggests that mitochondrial biogenesis is not a one-way program but a dynamic feedback system. Beyond responding to immediate energy demand or stress, mitonuclear signaling may allow cells to anticipate future metabolic needs, adjusting ribosome assembly, translation efficiency, and mtDNA replication in a predictive manner. Furthermore, integration with other cellular signaling networks—such as nutrient-sensing mTOR pathways, hypoxia-inducible factors may fine-tune this system, ensuring that mitochondrial capacity and cellular energy metabolism are optimally aligned with both environmental cues and internal physiological state.

### Role of RNA-binding proteins in maintaining translational homeostasis

Maintaining proper stoichiometry of respiratory chain components under homeostasis requires remarkable coordination between mitochondrial and nuclear genomes. Although the synthesis rates of mitochondrial subunits can vary more than 30-fold, cells achieve a functional balance that prevents the accumulation of orphan or misassembled proteins. This balance depends on RNA-binding proteins that stabilize transcripts and modulate their translation. LRPPRC, for example, promotes the stability and translation of *COX1* and *COX3* mRNAs and has been implicated in coordinating mitochondrial protein synthesis with nuclear gene-expression programs. Similarly, the translational activator TACO1 selectively enhances COX1 translation, and its impairment leads to reduced COX1 protein levels and defective complex IV assembly even when mRNA abundance remains normal (Fig. 2). CLUH (Clustered Mitochondria Homolog) further contributes by binding nuclear-encoded mitochondrial mRNAs and regulating their stability and localization, ensuring coordinated translation of proteins required for mitochondrial biogenesis and energy production (Fig. 3).

RNA-binding proteins (RBPs) are post-transcriptional regulators that modulate the fate of mRNAs and mitochondrial translation (39). In mitochondria, RBPs are involved in RNA stabilization, processing, maturation, and translation. Some RBPs associate with mitoribosomes, while others function independently to regulate RNA metabolism (51). Several mitoribosome-associated RBPs are crucial for ribosome structure and assembly. For instance, MRPS24, MRPS27, MRPL1, and MRPL12 serve as structural components that stabilize and bind mitochondrial rRNA, ensuring proper assembly and function of the mitoribosome (52). Nuclear-encoded factors such as C4orf14 and C6orf203 are imported into mitochondria, where they act as assembly factors facilitating ribosome maturation (53) (Table 3). Their activity helps modulate mitochondrial gene expression and energy production in response to cellular conditions. Defects in RBPs can lead to impaired mitochondrial translation, resulting in a deficiency in energy metabolism. They are known to cause a range of diseases, such as neurodegenerative disorders, metabolic syndromes, and mitochondrial encephalopathies.

Importantly, RBPs may also act as dynamic sensors that link mitochondrial translation to cellular metabolic status, adjusting ribosome assembly or mRNA stability in response to changes in ATP levels, redox state, or nutrient availability. This suggests that beyond maintaining homeostasis, RBPs provide a flexible regulatory network that allows mitochondria to rapidly adapt protein synthesis to both physiological and stress conditions, potentially coordinating with signaling pathways such as the integrated stress response to preserve cellular energy balance.

### Activation of the mitochondrial unfolded protein response (mtUPR) and integration with the cellular integrated stress response (ISR) and mito-dynamics

One of the most important mitochondrial stress responses is the mitochondrial unfolded protein response (mtUPR). Triggered by misfolded proteins, impaired translation, or OXPHOS defects, the mtUPR reduces mitochondrial burden by shifting metabolism toward glycolysis and amino-acid catabolism while downregulating TCA cycle activity and OXPHOS gene expression. It also alters mitochondrial RNA metabolism, as seen with the suppression of MRPP3, a subunit of mitochondrial RNase P, which leads to defective transcript processing and attenuated translation (54). Similar perturbations, such as MRPS5 deficiency, induce mito-nuclear imbalance and activate mtUPR, linking translation stress with nuclear compensatory pathways (55).

These stress-responsive pathways also influence cytosolic proteostasis. Mitochondrial import defects, often resulting from impaired mito-translation, lead to the accumulation of un-imported precursor proteins in the cytosol. This activates the UPR, which enhances proteasomal degradation to prevent toxic aggregation. Simultaneously, mitochondrial chaperones and proteases - including HSP60, HSP10, mtHsp70, LONP1, and CLPP are upregulated to restore mitochondrial protein homeostasis. These adaptations have broader physiological consequences; for example, T-cell activation and effector cytokine production decline when mitochondrial and cytosolic translation are suppressed during stress.

Because most mitochondrial proteins are synthesized in the cytosol, disruptions in mitochondrial translation also affect cytosolic proteostasis. Mutations that impair mitoribosome function increase protein aggregation and ROS production, whereas enhanced translational accuracy reduces proteostatic burden and modulates cellular aging. mTORC1 further integrates signals from mitochondrial translation status by adjusting the synthesis of nuclear-encoded mitochondrial proteins. Under conditions such as mtDNA replication defects, mTORC1 becomes aberrantly activated and stimulates ATF4-dependent mitochondrial stress responses, augmenting mtUPR activity and promoting secretion of metabolic cytokines such as FGF21. Recent work also suggests that mTORC1 adjusts cytosolic translation in response to mitochondrial translation defects such as mtEF4 loss, reinforcing its role as a central regulator of mitochondrial-cytosolic communication (56).

Mitochondrial stress also engages the integrated stress response (ISR), which serves as a broader cytoprotective mechanism. Stress-activated kinases, including GCN2, PERK, and HRI, phosphorylate eIF2α, resulting in a global reduction in cap-dependent translation while allowing selective translation of transcripts such as ATF4. ATF5, the mammalian counterpart of ATFS-1 in *C. elegans*, contributes to this response by inducing protective gene expression and is elevated in individuals with mitochondrial disorders. The OMA1-DELE1-HRI axis represents an additional mitochondrial branch of the ISR: upon stress, OMA1 cleaves DELE1, allowing it to accumulate in the cytosol and activate HRI, activating ATF4-dependent response (57) (Fig. 3).

Mitochondrial dynamics, comprising the balance of fusion, fission, motility, and mitophagy, acts as an essential upstream modulator and downstream effector of both the mtUPR and ISR. Under translation stress or accumulation of misfolded proteins, mitochondria frequently undergo protective fragmentation mediated by DRP1 activation. This fission response helps segregate damaged regions, facilitating selective mitophagy and preventing the propagation of dysfunctional mitochondrial nucleoids. In contrast, the fusion proteins OPA1 and MFN1/2 work less effectively during mtUPR activation (58). As a result, mitochondria fuse less, which keeps damaged parts of the mitochondria separated instead of mixing with healthy regions. Notably, OMA1 activation already central to the DELE1-HRI axis simultaneously cleaves OPA1, linking ISR initiation with structural remodeling of the mitochondrial network (59).

The mitochondrial dynamic changes are not merely structural but feedback into stress signaling. Fragmented mitochondria exhibit altered cristae architecture and reduced respiratory efficiency, which increases ATF4-driven metabolic rewiring toward glycolysis. Likewise, defects in fusion, such as MFN2 loss, exacerbate mito-nuclear communication errors and increases the mtUPR-related proteins, demonstrating that mitochondrial morphology directly shapes the magnitude and duration of stress responses. Intriguingly, recent studies suggest that enhanced mitophagy under conditions of translational imbalance reduces cytosolic proteotoxicity by clearing mitochondria overloaded with unassembled respiratory-chain proteins, thereby tightening the coupling between mitochondrial dynamics and cytosolic proteostasis (Fig. 3).

Taken together, these findings show that changes in mitochondrial shape act as a regulator that adjusts how strongly the mitochondrial stress response (mtUPR) and the integrated stress response (ISR) are activated. Import defects trigger not only proteostatic responses but also architectural remodeling of the mitochondrial network, which in turn modulates metabolic flux, organelle turnover, and communication with the cytosol. The combined action of mTORC1 signaling, integrated-stress kinases, mtUPR transcriptional programs, and dynamic mitochondrial remodeling forms a multi-layered regulatory system that prioritizes cellular survival by coordinating mitochondrial efficiency, cytosolic translation, and global proteostasis. This integrative perspective highlights how mitochondria act not as isolated organelles but as central hubs of cellular stress adaptation, capable of orchestrating coordinated responses across subcellular compartments and influencing organismal physiology.

### Ribosome remodeling and stalling during metabolic stress

Recent findings indicate that mitoribosome remodeling occurs under metabolic stress, driving a powerful effect on mt-ribosome composition and function to meet the energy demands of the cell (60). Changes in nutrient availability, ATP/ADP ratio, redox balance, and levels of key metabolites such as NAD^+^, acetyl-CoA, and α-ketoglutarate lead to incorporation or modification of ribosomal proteins, post-translational modifications such as phosphorylation, acetylation, and succinylation, and the maturation of rRNA, resulting in specialized mitoribosome populations with altered translational capacity. This remodeling directly impacts mitochondrial translation fidelity, and the selective synthesis of oxidative phosphorylation (OXPHOS) subunits. This remodeling ensures that mitochondrial protein synthesis is tightly coordinated with metabolic flux, thereby optimizing oxidative phosphorylation efficiency and mitochondrial biogenesis. Metabolic stress conditions such as hypoxia, nutrient scarcity, or altered TCA-cycle flux modulate the activity of ribosome assembly factors, RNA helicases, and quality-control proteins, coordinating mitoribosome biogenesis with mitochondrial DNA transcription and protein import.

Published literature provide clear evidence that mitoribosome structure and function can change in response to metabolic cues and stress (60). In yeast, shifts in nutrient availability remodel mitoribosome assembly pathways, producing ribosomes with altered composition and translational output that match the metabolic state of the cell. In mammals, mitochondrial mistranslation is strongly influenced by metabolic stress, as shown in mice where a high-fat diet exacerbates defects in mitochondrial translation fidelity and leads to tissue-specific dysfunction. Additionally, recent work has demonstrated that human mitochondria use factors like TACO1 to resolve ribosome stalling, highlighting the dynamic regulation of mitoribosome behavior under physiological stress (61). Together, these published findings support the idea that mitoribosome remodeling is an adaptive mechanism that tunes mitochondrial translation to metabolic conditions.

This suggests that the composition and assembly of mitochondrial ribosomes (mitoribosomes) are not static-rather, they can be reconfigured depending on the cell’s metabolic environment or stress conditions. Under changes in nutrients, energy availability or mitochondrial stress, cells may remodel mitoribosome assembly pathways so that different ribosomal proteins or conformations are preferentially incorporated. This “remodeling” might mitochondrial translation activity to meet metabolic demand: for example, favoring synthesis of oxidative phosphorylation (OXPHOS) proteins when energy production is needed, or reducing translation when nutrients are scarce to conserve resources. In addition, such remodeling is suggested to feedback regulate overall mitochondrial biogenesis coordinating the translation of mitochondrially encoded proteins with import of nuclear-encoded mitochondrial proteins. Thus, mitoribosome remodeling offers a flexible, metabolism dependent mechanism to tune mitochondrial protein synthesis and energy production operating partly independently of canonical cytosolic signaling pathways.

## Impact of mitochondrial translation on mitochondria-mediated immune outcome

The immune system is composed of metabolically dynamic cells, including macrophages, T cells, B cells, and dendritic cells, which must adapt their energy metabolism to fulfill specialized functions. Immune activation, cytokine production, and cell polarization are energetically demanding processes that rely on finely tuned mitochondrial function. Mitochondria act as highly integrated hubs that couple cellular metabolism, redox homeostasis, and innate immune signaling, thereby exerting profound control over inflammatory outcomes. Their regulatory influence begins with mitochondrial reactive oxygen species (mtROS), which are generated as natural by-products of electron transport chain activity. Under basal conditions, mtROS function as controlled second messengers that help activate antimicrobial pathways. However, when mitochondrial stress or dysfunction disturbs electron transport, ROS levels rise dramatically, leading to oxidative damage of lipids, proteins, and mitochondrial membranes. This oxidative stress promotes the externalization of cardiolipin and the release of mitochondrial DNA (mtDNA) into the cytosol - events that serve as danger-associated molecular patterns (DAMPs). These mitochondrial DAMPs, together with elevated mtROS, amplify redox-sensitive transcriptional pathways such as NF-κB and AP-1, increase the transcription of pro-inflammatory cytokines, and provide essential triggers for activation of the NLRP3 inflammasome. Through this mechanism, damaged mitochondria act as upstream initiators of inflammation, linking energetic stress directly to the maturation of IL-1β and IL-18 and to the broader cytokine environment.

Cytokine production is further modulated by mitochondrial metabolism itself. The TCA cycle produces metabolites that act as potent immunomodulators: succinate accumulates under inflammatory conditions and stabilizes HIF-1α, driving IL-1β expression; citrate supports the synthesis of prostaglandins and nitric oxide; fumarate modifies proteins through succination and influences inflammatory gene transcription; and the immune-metabolite itaconate exerts anti-inflammatory effects by inhibiting succinate dehydrogenase (SDH) and activating NRF2-dependent antioxidant pathways. In this way, fluctuations in mitochondrial metabolism shape the transcriptional landscape of immune cells and integrate energy status with cytokine output.

These metabolic signals are tightly coupled to mitochondrial dynamics. Mitochondria constantly undergo fission and fusion, and the balance between these processes determines not only organelle morphology but also immunological behaviour. Enhanced fission, often mediated by DRP1, produces fragmented mitochondria that generate high levels of ROS and are strongly associated with pro-inflammatory states, such as in activated macrophages and effector T cells. Conversely, mitochondrial fusion, regulated by MFN1/2 and OPA1, promotes efficient oxidative phosphorylation, lowers ROS levels, and supports anti-inflammatory or regulatory phenotypes (62, 63, 64). Thus, the structural state of mitochondria acts as an early determinant of whether a cell shifts toward an inflammatory or a resolving response.

When mitochondria become irreversibly damaged, mitophagy emerges as a critical anti-inflammatory mechanism by selectively removing dysfunctional organelles. Through the PINK1-Parkin pathway and other receptors, mitophagy limits ROS accumulation and prevents further leakage of mtDNA and cardiolipin that would otherwise fuel inflammasome activation. Insufficient mitophagy, therefore, leads to persistence of damaged mitochondria, chronic activation of inflammatory pathways, and, eventually, the development of sterile inflammatory diseases. Before mitophagy is engaged, mitochondrial proteostasis is maintained through the mitochondrial unfolded protein response (mtUPR), which increases the production of chaperones and proteases in response to protein-folding stress. A successfully resolved UPR restores mitochondrial function and dampens inflammatory signaling, whereas prolonged or unresolved mtUPR can paradoxically promote inflammatory transcriptional programs, acting as a cellular alarm state that maintains cytokine production.

Beyond these internal stress-response systems, mitochondria serve as the command center for innate immune pathways. The mitochondrial antiviral signaling protein MAVS, located on the outer mitochondrial membrane and enriched at mitochondria-ER contact sites, forms a scaffold for RIG-I and MDA5 signaling, leading to IRF3 and NF-κB activation and subsequent interferon and cytokine production during viral infection. Likewise, mtDNA that escapes into the cytosol activates the cGAS-STING pathway, driving type I interferon responses, while mitochondrial RNA, including double-stranded RNA generated during transcription, are recognized by TLR7/8 in endosomes or cytosolic RIG-I-like receptors, creating a sterile “virus-mimicking” inflammatory signal. These nucleic acid-based pathways demonstrate how mitochondrial integrity is continuously altered by the innate immune system.

**Figure 4:**
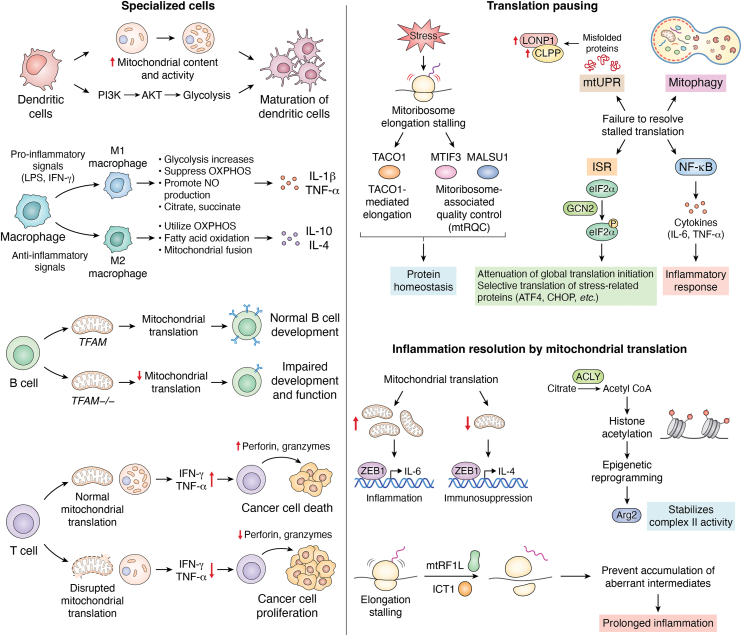
**Mitochondrial translation in specialized immune cell and its regulation through adaptive mechanisms of translational pausing and resolution-phase reprogramming.***A*, mitochondrial translation in specialized immune cell: Immune cells dynamically regulate mitochondrial translation to position with their metabolic and functional demands. During maturation, dendritic cells (DCs) increase their mitochondrial content supported by increased glycolysis *via* PI3K-AKT signaling. Pro-inflammatory M1 macrophages suppress mitochondrial translation and oxidative phosphorylation (OXPHOS), favoring glycolysis, whereas anti-inflammatory M2 macrophages promote OXPHOS through enhanced mitochondrial translation. B cells also modulate mitochondrial translation to support activation, antigen processing, and adaptive immune responses. Loss of TFAM reduces mitochondrial translation and impairs B-cell development. In T cells, disrupted mitochondrial translation impairs cytokine production and weakens inflammatory responses against cancer, affecting both cell proliferation and cytotoxicity. *B*, Translational pausing: Under stress conditions, mitoribosomes are prone to stalling. Mitochondrial translation factors such as TACO1 and ICT1 help resolve these pauses and maintain the integrity of the respiratory chain. Ribosome stalling activates quality control mechanisms including mitoribosome-associated quality control (mtRQC), mitophagy, and the mitochondrial unfolded protein response (mtUPR) to preserve mitochondrial homeostasis. Additionally, stalled mitoribosomes can trigger pro-inflammatory signaling pathways such as NF-κB and the integrated stress response (ISR), which attenuate global protein synthesis while selectively upregulating stress-related proteins like ATF4 and CHOP to support inflammation resolution. *C*, inflammation resolution by mitochondrial translation: The transcription factor Zeb1 inhibits macrophage repolarization from the pro-inflammatory M1 state to the anti-inflammatory M2 phenotype by modulating mitochondrial metabolism, thus preventing prolonged immune activation. Ribosome rescue pathways involving mitochondrial release factors (eg: mtRF1L) ensure translational fidelity under stress conditions. Furthermore, cytokines influence epigenetic reprogramming by modulating metabolic intermediates such as citrate and acetyl-CoA, which promote histone acetylation. Enzymes like Arginase 2 (Arg2) stabilize OXPHOS and support metabolic recovery, contributing in the resolution of inflammation and restoration of immune homeostasis.

These signaling processes are further coordinated by the physical architecture of the cell. Mitochondria maintain close contact with other organelles, and these contact sites profoundly shape inflammatory responses. ER-mitochondria contact points, or MAMs, regulate calcium transfer, lipid synthesis, and MAVS-dependent antiviral signaling; they also serve as platforms for inflammasome assembly. In the 2024 study, Sharma *et al.* (7) demonstrated that under inflammatory conditions, ORMDL3 localizes to ER-mitochondria contact sites, modulates mitochondrial morphology and fission, and thereby enhances NLRP3 inflammasome activation and IL-1β release, highlighting a key link between mitochondrial dynamics and NLRP3-driven inflammation. Mitochondria-lysosome interactions orchestrate the initiation of mitophagy and thereby determine the threshold for inflammasome activation. Interplay with peroxisomes further modulates antiviral responses by influencing ROS metabolism and interferon signaling. Through these organelle networks, mitochondria integrate metabolic, ionic, and structural cues into a coordinated inflammatory response (Fig. 4).

Ultimately, the influence of mitochondria on inflammation is most evident in immune cell differentiation and function. In macrophages, inflammatory polarization is tightly controlled by mitochondrial metabolism: M1 macrophages exhibit disrupted TCA cycle activity, increased succinate and mtROS, and a shift toward glycolysis, all of which promote production of IL-1β, IL-6, TNF-α, and nitric oxide. In contrast, M2 macrophages rely on fatty acid oxidation, intact oxidative phosphorylation, and low ROS, enabling them to produce anti-inflammatory cytokines such as IL-10. T lymphocytes display similar mitochondrial dependencies. Activated effector T cells adopt fragmented mitochondria, high glycolytic flux, and ROS signaling to sustain rapid cytokine production, whereas regulatory T cells (Tregs) and memory T cells depend on fused mitochondria, robust oxidative phosphorylation, and metabolic flexibility to maintain long-term survival and controlled immune responses. Through this metabolic and structural reprogramming, mitochondria not only dictate immediate inflammatory signaling but also shape long-term immune memory, tolerance, and tissue homeostasis.

Taken together, mitochondrial ROS generation, TCA cycle-derived metabolites, nucleic acid release, antiviral signaling platforms, organelle contact dynamics, quality-control pathways such as mitophagy and mtUPR, and cell-type-specific metabolic programming all converge to position mitochondria as master regulators of inflammation. Their ability to sense stress, integrate diverse biochemical signals, and orchestrate innate and adaptive immune responses makes mitochondrial health a defining determinant of inflammatory balance, host defence, and the progression or resolution of immune-mediated diseases.

Mitochondrial translation is not an isolated biochemical process but a central determinant of mitochondrial health. When translation proceeds normally, mitochondria sustain optimal OXPHOS, low ROS, balanced dynamics, stable mtDNA, efficient mitophagy, healthy organelle contacts, and appropriate antiviral signaling—all of which maintain inflammatory homeostasis. When translation deteriorates, mitochondrial stress rapidly spreads through redox imbalance, proteostasis failure, metabolic rewiring, and innate immune activation, ultimately producing exaggerated cytokine responses, enhanced inflammasome activity, and disrupted immune-cell differentiation. Therefore, mitochondrial translation represents a fundamental upstream regulator that integrates mitochondrial function with cellular inflammatory fate.

Defects in mitochondrial translation, such as disrupted mito-ribosome assembly, mutations in mitochondrial tRNAs, or loss of translation factors—result in immune activation. Impaired translation compromises the integrity of the electron transport chain, leading to electron leakage, enhanced ROS formation, membrane depolarization, and ultimately the release of mtDNA into the cytosol. Once in the cytosol, mtDNA acts as a potent DAMP that activates cGAS, triggering the cGAS-STING-TBK1-IRF3 axis and inducing NF-κB signaling and type I interferon responses. Studies also showed that TFAM depletion increases mtDNA leakage and activates cGAS-STING-dependent interferon signaling in hepatocytes (65). Similar mtDNA-driven activation of STING has also been shown in macrophage-driven inflammation during atherosclerosis.

### Immune activation triggered by mitochondrial translation defects

Mitochondrial stress also increases the cytosolic presence of mtRNA, activating RIG-I-like receptors and TLR7, which further amplify inflammatory responses. For instance, in Irgm1-deficient mice, fibroblasts primarily sense mtDNA through cGAS-STING, whereas macrophages detect mtRNA *via* TLR7, illustrating cell-type-specific PRR preferences (66). Oxidized mtDNA is particularly effective at activating the NLRP3 inflammasome, leading to IL-1β and IL-18 secretion and metabolic reprogramming toward glycolysis and lipogenesis. Numerous factors, including mitochondrial micropeptides, membrane permeability regulators such as VDAC, and pharmacological modulators like rotenone, shape these inflammatory outcomes.

Mitophagy normally restricts this inflammatory response by clearing damaged mitochondria and their genetic material. It has been found that Parkin deficiency, impaired mitophagy, allows mtDNA to accumulate, heightening systemic inflammation. Although mitochondrial-derived vesicles (MDVs) represent an additional quality-control pathway that transports damaged mitochondrial components to lysosomes, severe mitochondrial translation defects can increase MDV formation and intensify DAMP-mediated activation. Despite these advances, fundamental questions remain regarding why certain immune cells are more sensitive to mitochondrial DAMPs and whether distinct translation defects generate unique immune activation signatures (Fig. 4).

### Specialized mitochondrial translation programs in immune cells

Immune cells have specialized mitochondrial translation patterns adapted to their roles. Macrophages offer a clear example of this specialization. Pro-inflammatory M1 macrophages rely on aerobic glycolysis and downregulate OXPHOS and mitochondrial translation, accumulating intermediates such as succinate and citrate that support inflammatory signaling. In contrast, anti-inflammatory M2 macrophages depend on OXPHOS activity and correspondingly upregulate mitochondrial translation, mitochondrial biogenesis, and dynamic remodeling of the mitochondrial network. Cytokines like IL-25 enhance mitochondrial ATP production to support M2 polarization, whereas tetracycline-mediated inhibition of mitochondrial translation dampens LPS-induced cytokine production, making mito-translation a vital mechanism to maintain macrophage activation states (67, 68).

T and B lymphocytes further demonstrate how mitochondrial translation is adapted to immune specialization. CD8^+^ T cells with mtDNA mutations acquired from tumors, display impaired OXPHOS and reduced effector function, whereas thermal stress restores mitochondrial pathways and enhances their cytotoxic capacity. B cells increase mitochondrial transcription and translation during antibody diversification, and cytotoxic T lymphocytes expand their mitotranslation capacity to maintain sustained effector function. The cytokines also shape mitochondrial metabolism in T cells: IL-27 restricts OXPHOS and mitochondrial biogenesis to limit the expansion of Th1 and Th17 cells, while IL-10 preserves OXPHOS by increasing nitric oxide signaling and mTOR activity. IL-4 promotes OXPHOS-driven Acetyl-CoA production necessary for epigenetic activation of anti-inflammatory gene programs. Dendritic cells likewise enhance their mitochondrial translation and respiration during maturation, enabling efficient antigen presentation and T cell priming (Fig. 4).

Collectively, these observations demonstrate that mitochondrial translation is not a simple metabolic process but an essential determinant of immune cell identity and function. By controlling mitochondrial protein synthesis, cells adjust their bioenergetic and signaling capacities to perform immunological functions.

### Mitochondrial translational pausing and mitoribosome stalling as immune modulators

Translational pausing within mitochondria has emerged as an important bridge connecting mitochondrial proteostasis with immune signaling. Under conditions of metabolic stress or during the decoding of difficult sequences such as polyproline tracts, mitoribosomes can stall. Factors like TACO1 help resolve such stalling events by stabilizing the peptidyl transferase centre and sustaining translation of critical respiratory chain components. When pausing persists, mitochondria activate mitoribosome-associated quality-control (mtRQC) pathways, which recruit rescue factors to release stalled ribosomes and maintain protein homeostasis.

Prolonged or unresolved stalling activates the mitochondrial unfolded protein response (mtUPR), leading to the upregulation of mitochondrial chaperones, proteases, and regulators to resolve proteotoxic stress. While mtUPR activation is protective in transient stress, such as during inflammatory bowel disease but chronic activation can worsen tissue damage, as demonstrated in models of pulmonary fibrosis and autoimmune dermatitis. Ribosomal stalling also promotes mitophagy, thereby preventing the accumulation of dysfunctional mitochondria and the unnecessary release of mtDAMPs. In parallel, stalling induced by oxidative stress can activate the GCN2-eIF2α arm of the integrated stress response, influencing global translation and innate immune sensitivity. Disruptions in mitochondrial dynamics, particularly excessive fission, amplify ROS production, hinder mitochondrial translation, and create positive-feedback loops that increase inflammatory signaling (Fig. 4).

Moreover, fragmented mitochondria common during hypoxia and inflammation, are prone to releasing mtDNA and mtRNA, making them inherently immunogenic. In such conditions, stalled ribosomes can activate NF-κB and drive pro-inflammatory cytokine production, especially when mitophagy is impaired. Recent studies also indicate that mitochondrial translational pausing may function as an immunogenic stress signal with potential relevance across chronic diseases ranging from IBD and NASH to neurodegenerative disorders (69).

### Resolution of inflammation through mitochondrial translation and cytokine signaling

Mitochondrial translation not only initiates immune activation but also plays a pivotal role in resolving inflammation. A hallmark of this resolution phase is the transition of macrophages from the pro-inflammatory M1 state to the tissue-repairing M2 phenotype. This shift depends on mitochondrial rewiring driven by regulators such as ZEB1, whose activation is promoted by agents like metformin, which reduces inflammation, whereas ZEB1 deficiency results in exaggerated immune responses (70). Nitric oxide produced by M1 macrophages inhibits OXPHOS and mitochondrial translation; its suppression enhances mitochondrial respiration and supports M2 repolarization. In this context, ribosome rescue pathways become crucial: ICT1, a mitochondrial release factor, resolves non-stop mitoribosome stalling events and prevents the accumulation of aberrant translational intermediates that could prolong inflammation. Similarly, mtRF1L and other rescue factors preserve mitochondrial translational fidelity under stress and support the transition toward a resolution phenotype.

Anti-inflammatory cytokines such as IL-10 and IL-4 further stabilize mitochondrial function during the resolution phase. IL-10 maintains OXPHOS activity by reducing nitric oxide levels or inhibiting mTOR signaling (33), while IL-4 enhances acetyl-CoA production and histone acetylation through enzymes such as Arg2 to promote anti-inflammatory transcriptional programs (71). Additional pro-resolving processes, including mitochondrial transfer from mesenchymal stem cells to immune cells, restore bioenergetics and support tissue repair. Specialized pro-resolving mediators (SPMs), including resolvins, shift cellular metabolism toward enhanced mitochondrial respiration while suppressing inflammatory gene expression (Fig. 4).

Perturbations in this metabolic-translational axis are increasingly recognized as drivers of chronic immune disorders, including chronic airway inflammation, chronic kidney disease, type 2 diabetes, inflammatory bowel disease and rheumatoid arthritis, which we will explain in next section.

## Mitochondrial translation defects in inflammatory disease

**Figure 5:**
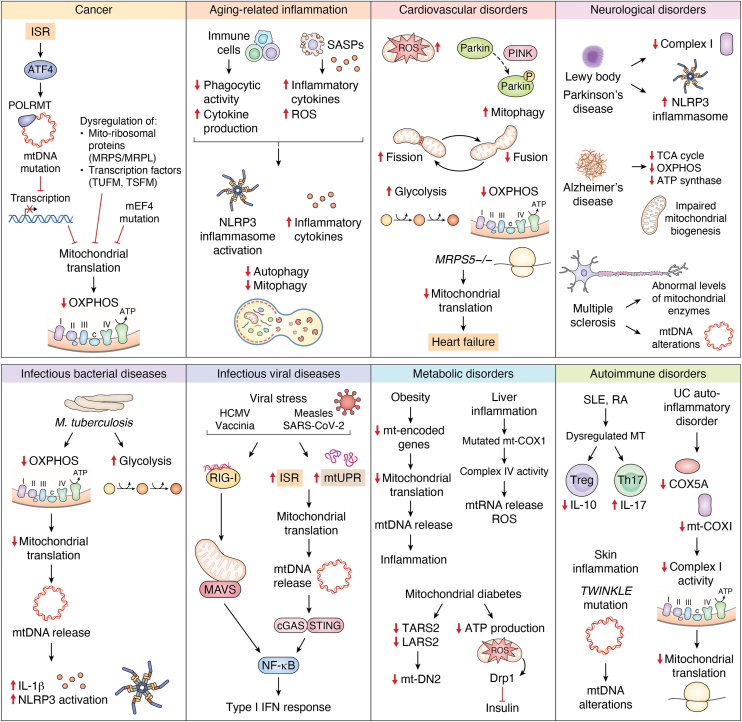
**Mitochondrial translation dysregulation in disease pathogenesis.** This figure highlights the clinical pathologies arising from defects in the mitoribosomal proteins, mtDNA mutations, or translation factors contribute to diverse inflammatory and degenerative disorders. Cancer: Mutations in mEF4 suppress mitochondrial translation and OXPHOS, promoting tumor growth. Enhanced POLRMT expression and mtDNA mutations activate ISR signaling, while mitotranslation inhibition induces apoptosis and therapeutic vulnerability. Aging-related inflammation: Reduced translation fidelity increases ROS, SASP expression, and NLRP3 inflammasome activation, driving chronic inflammation with impaired autophagy and mitophagy. Cardiovascular Disorders: Loss of MRPS5 impairs mitochondrial translation, elevates glycolysis, and disrupts mitochondrial dynamics, contributing to cardiac dysfunction. Neurological Disorders: mtDNA alterations and defective mitotranslation are linked to Parkinson’s, Alzheimer’s, and multiple sclerosis *via* inflammasome activation, impaired enzyme levels, and disrupted OXPHOS and TCA cycle function. Infectious Bacterial Diseases: Bacteria (*e.g.*, *M. tuberculosis*) - inhibits OXPHOS, shift to glycolysis, and activate NLRP3 *via* mtDNA release. Infectious Viral Diseases: Viruses (*e.g.*, HCMV, SARS-CoV 2): Hijack mitochondrial translation through MAVS/RIG-I and trigger ISR. This leads to mtDNA release and cGAS-STING activation, promoting type I IFN response. Metabolic Disorders: Obesity- Dysregulated mt-encoded gene translation drives inflammation *via* mtDNA release. Liver Inflammation- Mutated mt-COX1 reduces complex IV activity and translation, promoting ROS and inflammation. Mitochondrial Diabetes - TARS2/LARS2 and ND2 downregulation impair ATP synthesis; Drp1 activation disrupts insulin signaling. Autoimmune Disorders: SLE & RA - Defective translation alters Treg/Th17 balance, promoting inflammation. Ulcerative Colitis - Impaired complex I and OXPHOS from COX5A and mt-COX1 downregulation reduce translation efficiency. Skin Inflammation - TWINKLE helicase mutations lead to mtDNA instability and defective mitochondrial translation.

We will next describe the effects of altered mitochondrial translation on various immune diseases and also diseases where inflammation dictates pathology as depicted in Figure 5.

### Cancer

Mitochondrial translation plays a critical role in sustaining tumor growth, immune evasion, and adaptation to stress. Tumor cells often rely on mitochondrial energy remodeling to survive in hypoxic and nutrient-depleted microenvironments. Dysregulation of mitochondrial ribosomal proteins (MRPs), aberrant OXPHOS activity, and mtDNA mutations or copy number alterations have been implicated in various cancers, while mitoribosome inhibition suppresses proliferation and survival (72, 73, 74). For instance, Ishikawa *et al.* demonstrated that mutations in mitochondrial NADH dehydrogenase genes impair respiratory chain activity and increase ROS production, promoting genetic instability and tumorigenesis (75).

Increased expression of POLRMT, the mitochondrial RNA polymerase, is frequently observed in solid tumors and hematologic malignancies. It enhances mitochondrial biogenesis and supports the metabolic demands of rapidly proliferating cancer cells. These changes not only drive tumor progression but also influence the tumor immune microenvironment. Accumulation of mitochondrial ROS and release of mitochondrial DAMPs, such as mtDNA, can shape anti-tumor immunity by activating innate immune pathways or, in contrast, by inducing immunosuppressive responses under chronic stress conditions (76).

Recent studies also reveal that mitochondrial translation stress triggers adaptive responses that support immune escape and resistance to apoptosis (77). Tumors undergoing mitochondrial stress can activate the integrated stress response (ISR), enabling them to tolerate chemotherapy or immune attack (78). In this context, mitochondrial translation inhibitors have emerged as potential adjuvants to overcome therapy resistance. For example, targeting mitochondrial translation sensitizes aggressive myeloid leukemias to venetoclax, a BCL2 inhibitor, enhancing apoptotic priming (79). These studies suggest that dysregulation of mitochondrial translation machinery, including mitoribosomal proteins (MRPLs/MRPSs) and translation factors such as TUFM and TSFM, contributes to impaired oxidative phosphorylation, metabolic reprogramming, and enhanced tumor growth across multiple cancer types (10) (Fig. 5).

Also, MT stress activates ISR program, which enables tumor cells to survive apoptotic stimuli and evade immune detection. Thus, classifying tumors based on their mito-translation dependency could guide personalized therapy. Further investigation into the role of mitochondrial translation in cancer metastasis, immune evasion, and drug resistance will open new avenues for therapeutic intervention. Some chemoresistant tumors also retain dependence on mitochondrial translation to survive drug-induced stress, suggesting a selective vulnerability.

### Infectious diseases

Host-pathogen interactions, particularly in viral infections, often involve hijacking the host’s translational machinery for protein synthesis and survival. Beyond cytosolic translation, mitochondrial translation is now emerging as a critical interface shaping cellular metabolism and innate immune responses. Many viruses manipulate mitochondrial dynamics and function to optimize replication while evading immune detection. Interestingly, viruses like human cytomegalovirus (HCMV) and vaccinia virus selectively upregulate mitochondrial translation even during global host translation shutdown, thereby maintaining the bioenergetic support required for viral replication (80, 81). Disruption of mitoribosome biogenesis impairs viral propagation, further emphasizing its necessity (82).

Viral infections further exacerbate neuroinflammation *via* mitochondrial disruption. Viruses such as Zika and rabies hijack ETC subunits and interfere with OXPHOS to promote replication and induce ROS generation (83, 84). These perturbations compromise mitochondrial function, activate inflammasome pathways, and contribute to neuro-inflammatory pathology (85).

In addition to metabolic hijacking, viruses directly target mitochondrial components. For instance, SARS-CoV-2 causes its polypeptides, M and N proteins, to bind to mitochondrial tRNA synthetases and Complex I subunits, disrupting mitotranslation, triggering mtUPR and the ISR (86). These stress pathways modulate immune outputs and can blunt antiviral signaling (Fig. 5).

Importantly, mitochondrial translation defects can also enhance innate immune sensing. Viral stress often leads to the release of mitochondrial nucleic acids, including mtRNA, into the cytosol. Double-stranded mtRNA is recognized by RIG-I-like receptors (RIG-I, MDA5), which signal *via* MAVS, to activate NF-κB and Type 1 IFN-responses (87). This positions mitochondrial translation fidelity as a regulator of antiviral immunity ensuring that mitotranslation defects don’t lead to inappropriate immune activation or immune evasion. Pathogens may also alter mitochondrial translation indirectly. For example, measles virus induces mitochondrial hyperfusion and cytosolic mtDNA release, activating cytosolic DNA sensors (88). Similarly, infections like *Mycobacterium tuberculosis* rewire host metabolism, shifting from OXPHOS to glycolysis to change IL-1β production and inflammasome activation (89), though the precise role of mito-translation in this shift remains to be fully elucidated.

Overall, mitochondrial translation serves as a dual regulator in infectious diseases, *i.e.*, supporting viral replication under stress while also acting as a gatekeeper of antiviral immune signaling. Future work dissecting pathogen-specific alterations to mitochondrial translation could reveal new targets for immunomodulatory therapies.

### Metabolic & Autoimmune Diseases

Emerging evidence highlights mitochondria as critical regulators of immune tolerance and metabolic balance, with their dysfunction increasingly linked to chronic inflammatory conditions. In metabolic and autoimmune diseases, disruptions in mitochondrial protein synthesis impair cellular energetics, elevate ROS production, and release mitochondrial nucleic acids that act as potent triggers of inflammation.

In metabolic diseases like insulin resistance (IR), obesity, T2D, and diabetic kidney disease (DKD), mitochondrial translation defects hinder OXPHOS, ATP synthesis, and metabolic flexibility. For example, in mitochondrial diabetes, genetic defects in pancreatic β-cells lead to impaired ATP production and insulin secretion, disrupting glucose homeostasis (90). Mutations affecting COX1 translation, such as in COX14-deficient mice, result in liver inflammation through mtRNA release driven by ROS accumulation (91). Similarly, complex I defects reduce mitochondrial NADPH production, escalating inflammation and oxidative damage (92). In obese patients, mitochondrial dysfunction characterized by decreased mtDNA content, impaired fatty acid oxidation, and TCA cycle inhibition, contributes to adiposity and systemic inflammation (93). Suppression of mitochondrial aminoacyl-tRNA synthetases (mt-aaRSs), such as TARS2 and LARS2, in diabetic muscle impairs ND2 production and mitochondrial protein synthesis (94). These studies provide strong evidence that defective mitochondrial translation is causative in metabolic pathology and tissue inflammation (Fig. 5).

Notably, mitochondrial translation defects precede dysfunction in non-alcoholic fatty liver disease (NAFLD), showing defects occur at the initial stages of disease progression. Malik *et al.* showed that a high-fat diet in mice increases mtDNA without functional benefits, whereas a western diet causes mitochondrial dysfunction and mtDNA-driven inflammation, implicating role of translation failure in disease progression (95). Restoration of translation *via* AKAP1 knockdown improves mitochondrial membrane potential and gene expression in DKD, reinforcing the therapeutic potential of targeting mitochondrial translation (96).

In autoimmune diseases like rheumatoid arthritis (RA), systemic lupus erythematosus (SLE), and multiple sclerosis (MS), dysregulated mitochondrial translation exacerbates immune activation. T regulatory cells (Tregs), typically responsible for suppressing inflammation, become dysfunctional, allowing Th17 expansion as a key driver of autoimmunity (97). Hyperactive mitochondrial translation in autoantibody-producing B cells intensifies SLE pathology (98), while oxidized mtDNA and proteins act as neo-autoantigens in SLE and RA, eliciting aberrant immune responses (98). In RA, synovial fibroblasts show altered mito-translation, and in MS, neuronal mitochondrial dysfunction contributes to demyelination. Growing evidence shows that when mitochondrial function becomes dysregulated in immune cells, immune tolerance can break down, leading to heightened autoreactive responses. As a result, therapeutic strategies that target oxidative phosphorylation (OXPHOS) and electron transport chain (ETC) complexes, especially within T cells have emerged as promising approaches for reducing autoimmune activity (Fig. 5) (99).

Barrier organs like skin and intestine also depend on mitochondrial function for maintaining homeostasis and respond to environmental cues. Inflammatory bowel disease (IBD), particularly ulcerative colitis (UC), is associated with mitochondrial dysfunction in the intestinal epithelium, marked by reduced Complex I activity and impaired OXPHOS (100). Transcriptomic analysis in UC patients reveals downregulation of TCA cycle genes, and decreased expression of both mitochondrial-encoded (MT-CO1) and nuclear-encoded (COX5A) OXPHOS components (100). Multi-omics approaches have identified PARK7, FIS1, and PDK1 as IBD risk genes, implicating mitochondrial dynamics and translation defect in disease pathogenesis (101). In skin inflammation, mitochondrial dysfunction exacerbates conditions like psoriasis and atopic dermatitis. Mice with mutant TWINKLE helicase accumulate mtDNA deletions, leading to severe skin inflammation (102). Inhibiting mitochondrial translation using linezolid suppresses IL-17A^+^ γδ T cell responses and alleviates psoriasis severity (103). PRR activation in barrier tissues further amplifies inflammation by destabilizing mitochondrial complex I in an NLRP3- and ROS-dependent manner, enhancing IL-1β production. Additionally, COPD pathogenesis involves mitochondrial translation-related genes like MRPL2 and NDUFS2. NDUFS2 is upregulated in pulmonary macrophages and contributes to inflammation and impaired macrophage function (104). These findings present that mitochondrial translation is a critical regulator of local immune responses.

Collectively, these findings underscore mitochondrial translation as both a driver of inflammation and a key regulator of immune tolerance, especially during chronic stress. Defects in mitochondrial translation seem to be primary drivers of inflammation and immune dysfunction rather than secondary consequences in various inflammatory-metabolic diseases. In fact, defective mitochondrial translation serves as an early indicator that drives metabolic pathology, thereby offering a promising target for treating chronic inflammatory and autoimmune diseases.

### Cardiovascular Disorders & Neuro-inflammation

Both the heart and the brain are energy-intensive organs that rely heavily on mitochondrial oxidative phosphorylation (OXPHOS) for maintaining physiological function. Defects in mitochondrial translation compromise OXPHOS and ATP production, leading to metabolic collapse, increased oxidative stress, and ultimately inflammation-driven pathology (Fig. 5).

In the heart, more than 50% of individuals with mutations in mitochondrial protein-encoding genes develop some form of cardiomyopathy (105). During cardiac hypertrophy, significant changes in mtDNA-encoded gene expression and mitochondrial function are observed (106). Recent study shows that loss of mitochondrial ribosomal protein S5 (MRPS5/uS5m) results in cardiac hypertrophy and heart failure (55). Mechanistically, MRPS5 disruption reprograms mitochondrial translation, increases glycolysis, and reduces OXPHOS, ultimately impairing cardiac function (107). Despite its importance, how mitochondrial translation adapts during stress conditions such as ischemia, pressure overload, or metabolic shifts remains poorly understood.

Similarly, in the brain, mitochondrial translation defects drive neuro-inflammation and neuronal degeneration. Neurons are highly dependent on OXPHOS-derived ATP due to the absence of glycogen stores (108). Mitochondrial translation failure leads to ETC complex deficiency, ATP depletion, and synaptic dysfunction, often culminating in cell death. Inherited mitochondrial disorders such as Leigh syndrome and NARP (Neuropathy, Ataxia, and Retinitis Pigmentosa) are linked to mutations in mtDNA or nDNA encoded mitochondrial genes and result in severe, progressive neurological symptoms. A recently identified pathogenic MT-TY gene, which is located in the mitochondrial DNA and encodes for mitochondrial transfer RNA (mt-tRNA) for the amino acid tyrosine (109). The MT-TY gene variant (m.5889A>G) is associated with a severe multisystem childhood-onset disorder marked by neurodegeneration, seizures, and disrupted protein assembly, highlighting the role of mitochondrial translation failure (109).

Beyond primary mitochondrial disorders, mitochondrial dysfunction is implicated in neurodegenerative diseases such as Alzheimer’s Disease (AD), Parkinson’s Disease (PD), and amyotrophic lateral sclerosis (ALS). OXPHOS deficiency, often referred to as secondary mitochondrial dysfunction, is a hallmark of these diseases. In PD, mitochondrial dysfunction enhances NLRP3 inflammasome activation, promoting neuro-inflammation (110). Notably, NLRP3-deficient mice are protected from age-related cognitive and memory decline (111). In AD, mitochondrial translation inhibition has been shown to reduce cholesterol metabolism by downregulating SREBP2 and sterol biosynthesis genes, suggesting a novel mechanism underlying neuronal demyelination and dysfunction (112). Similarly, stroke-induced behavioral deficits are associated with reduced expression of mitochondrial proteins and mtDNA in perilesional cortical tissue (84, 113).

Therefore, these findings together identify mitochondrial translation as essential for both heart and brain, with its disruption driving inflammation and degeneration, making it a promising target to protect energy-demanding tissues under stress.

### Aging-related inflammation

A chronic, low-grade inflammation that progressively develops with age and contributes to the pathogenesis of multiple age-related diseases. Mitochondrial dysfunction is a central driver of this process, and emerging evidence implicates defective mitochondrial translation as a key driver of this pro-inflammatory aging phenotype (Fig. 5).

Reduced protein synthesis, including mitochondrial-encoded proteins, has been linked to increased lifespan in diverse model organisms, suggesting a tightly regulated link between translation and longevity (114). Conversely, mutations in mito-ribosomal DNA that reduce translational fidelity significantly shorten chronological lifespan, likely due to impaired expression of respiratory complexes and disrupted cellular homeostasis (115, 116). This is paralleled by alterations in nutrient-sensing and stress-responsive pathways such as TOR and Msn2/4 (Multi-copy Suppressor of SNF1 2/4), both of which influence aging and inflammation.

Impairment of mitochondrial translation activates stress signaling cascades, including the ATF4 pathway that regulates genes involved in oxidative stress adaptation, protein folding, and inflammatory cytokine production (117). ATF4 also promotes the transcription of senescence-associated secretory phenotype (SASP) genes, linking mitochondrial dysfunction to age-related cellular senescence and systemic inflammation. Simultaneously, aging cells exhibit reduced mitophagy, resulting in the accumulation of dysfunctional mitochondria and heightened immune activation (118). In immune cells such as macrophages and T cells, mito-translational stress impairs metabolic flexibility, contributing to immune-senescence and the decline of effective immune regulation in aged individuals (119). Notably, failure to resolve translational stalling in aging tissues chronically activates the mtUPR, which further reinforces inflammatory gene programs and tissue degeneration (120).

Mitochondrial translation accuracy appears to be tightly coupled with redox balance. While hypoaccurate translation promotes ROS accumulation and proteotoxic stress, enhanced accuracy of mitochondrial translation can attenuate ROS-induced protein aggregation and mitigate cellular aging phenotypes (121). This link between translation fidelity and longevity emerges as a breakthrough. More such studies focusing on mito-ribosomal fidelity and aging, extensive cytosolic and translational programs during age-related stress can provide a better overall understanding to mitigate the burden of age-related diseases.

## Pharmacological modulation of mitochondrial translation impacting inflammation

Pharmacological agents like particularly ribosome-targeting antibiotics and integrated stress response modulators can selectively alter mitochondrial protein synthesis, with therapeutic implications for inflammatory and metabolic diseases are discussed in detail below.

### Antibiotics as mitoribosome inhibitors

Mitochondrial ribosomes are susceptible to many antibiotics as they share similarities with bacterial ribosomes. To mention a few, ribosome-targeting antibiotics (RAbo) such as tetracycline, erythromycin and chloramphenicol selectively inhibit mitochondrial protein synthesis. RAbo binds the peptidyl transferase centre of the mitochondrial ribosome, interfering with the translation process. Another antibiotic in clinical use, Linezolid (an oxazolidinone antibiotic), inhibits mitochondrial protein synthesis by inhibiting mitochondrial cytochrome c oxidase (122, 123). Similarly, the next-generation oxazolidinone antibiotic, tedizolid, exhibits better efficacy than linezolid (124). Some other antibiotics used to alter mitochondrial translation in different diseases are mentioned in Table 4.

**Table 4:** Pharmacological modulators of mitochondrial translation

Antibiotics as Mitoribosome inhibitors		
**Compound**	**Target and Mechanism of Action**	**Immunological/Inflammatory Outcome Disease Relevance**
Tetracycline	Inhibits mitoribosome 30S subunit	Suppresses LPS-induced cytokines (IL-6, TNF-α) in macrophages (147)
Doxycycline	Binds 30S subunit, inhibits mitochondrial translation	Reduces inflammatory activation and cytokine release (148, 149)
Tigecycline	Blocks aminoacyl-tRNA entry at 30S ribosomal site	Induces mitochondrial dysfunction, reprograms metabolism in immune cells (150)
Linezolid	Inhibits mitoribosomal protein synthesis	Induces lactic acidosis and reduces OXPHOS in T cells and macrophages (123)


Compounds	Mechanism	Immunological Relevance
Integrated Stress Response (ISR) Modulators and Small Molecule		
Halofuginone	Activates ISR *via* mitochondrial remodeling	Enhances mitochondrial respiration, improves muscle-cell survival (125)
ISRIB	It inhibits eIF2alpha phosphorylation.	Restores translation during stress, protects neurons, reduces inflammation (151)
BtdCPU	Activates HRI - ATF4 *via* OMA1-DELE1 axis	Induces mtUPR; enhances stress resilience in immune cells (152)
Metabolic Modulators		
Rapamycin	Inhibits mTORC1 (mammalian target of rapamycin).	Altering cytosolic and mitochondrial translation; used in T-cell differentiation studies (153, 154, 155)
Metformin	Activates AMPK	Suppresses inflammatory cytokines in macrophages, Recent reports suggest links to mitochondrial complex I and mitotranslation. Acute respiratory distress syndrome-inhibits macrophages IL-1 beta and IL-6 production and abrogates acute respiratory distress syndrome. It inhibits the mitochondrial respiratory chain in liver (156)

### Integrated Stress Response (ISR) modulators and small molecules

ISR acts as a central hub for sensing mitochondrial dysfunction and regulating protein synthesis *via* eIF2α phosphorylation. Therapeutic targeting of mitochondrial pathways such as the mtUPR has emerged as a promising strategy to restore mitochondrial function under stress. Notably, ISR-activating compounds like BtdCPU and Halofuginone trigger mitochondrial remodeling and enhance the activity of the mitochondrial respiratory chain (125, 126). By modulating mitochondrial translation, proteostasis, and cellular stress sensing, these ISR-linked agents fine-tune immune responses, positioning them as valuable tools in inflammation-related disease modulation.

### Metabolic modulators

Some metabolic drugs, such as rapamycin and metformin, which impact mitochondrial translation indirectly *via* nutrient-sensing pathways such as mTOR or AMPK (127). Although they are not direct mitoribosomal inhibitors but can influence immune cell fate and inflammatory outcomes, as mentioned in Table 4.

Together, these pharmacological agents demonstrate how mitochondrial translation is not only a metabolic hub but also a regulatory axis for immune modulation. By targeting mito-ribosomal activity or ISR signaling, we can alter macrophage polarization, T cell activation, and inflammatory cytokine output.

## Discussion and future directions

Beyond merely producing components of the OXPHOS machinery, recent findings suggest that the mitochondrial translational apparatus is intimately involved in shaping immune cell fate, orchestrating inflammatory responses, and interfacing with broader cellular signaling networks. Defects in mitochondrial translation serve as potent triggers of innate immune activation through ROS and release of mitochondrial DAMPs (including mtDNA and oxidized mtRNA) that engage PRRs like the RIG-1, TLR7 and cGAS-STING. In immune cells, like macrophages, T-cells, B-cells and dendritic cells, mitochondrial translation is fine-tuned through metabolic shifts to meet lineage-specific demands, supporting various functions like cytokine production, antigen representation and immune memory. Recent studies revealed translational pausing and ribosome stalling, especially under oxidative stress can act as immune-regulatory signals linking mitochondrial perturbation to activation of the inflammasome, NF-κB, and ISR pathways. These findings deepen our understanding of the specialized stress-adaptive mechanisms employed by immune cells, however, the regulation of mitochondrial translation across diverse immune subsets such as naive, effector, and memory T cells, macrophage subsets, dendritic cells and B cells, remains poorly characterized. These studies also open new avenues for future research, raising questions about role of mitoribosome stalling merely as a cellular stress marker or that actively function to regulate downstream gene expression and determine cell fate. Moreover, the discovery of Mito-Neo-Peptides (MNPs), derived from stress-induced translational errors, strongly support that mitochondrial translation not only supports metabolism but acts as a source of immune-modulatory signal, possibly functioning as neoantigens or DAMPs during immune surveillance.

We also discussed how mitochondrial translation serves as a major regulator of immune response across a wide range of inflammatory disease progression including skin inflammation, IBD, cancer, metabolic syndrome, neurodegeneration, cardiovascular disease, and infections. For instance, viral and bacterial infections hijack mitochondrial translation to manipulate host immunity, similar to cancer cells, that exploit mitotranslational reprogramming for immune evasion and stress adaptation. However, in conditions like ulcerative colitis and psoriasis, downregulation of mitochondrial-encoded and nuclear-encoded OXPHOS genes impairs epithelial integrity and drives chronic inflammation. Also, in metabolic diseases such as NAFLD and diabetes, defects in translational machinery (eg: tRNA synthetases) distress mitochondrial energetics and amplify immune activation. Age-related inflammation and neurodegenerative diseases are marked by mitotranslational decline, mtDNA release, and activation of inflammatory cascades like the NLRP3 inflammasome and RIG-I pathway. Thus, defective mitochondrial translation is increasingly identified as early and causative events however, complete profile of disease-specific mitotranslational signatures is yet to be elucidated.

Conversely, mitochondrial translation has been shown in the resolution of inflammation through restoration of OXPHOS, enhanced translational fidelity, and activation of ribosome rescue mechanisms (*e.g.*, ICT1, mtRF1L) that support metabolic rewiring, immune tolerance, and macrophage repolarization. These findings add a new layer to our understanding of how mitochondrial translation fidelity may shape immune outcomes, suggesting it as a translation-centric immune checkpoint. Though the immunological consequences of translational fidelity errors (*e.g.*, amino acid misincorporations) have not been completely investigated. Also, exploring whether adjustments in mitochondrial translation to mitigate ribotoxic stress from immune activation to tolerance, particularly in chronic inflammatory diseases, can improve our understanding of immune regulation and tissue homeostasis, and its therapeutic potential in immune-mediated disorders.

Understanding mitochondrial translation requires linking molecular mechanisms with broader cellular and disease processes. One important direction is to explore how defects in mitoribosome assembly contribute not only to classical mitochondrial disorders but also to a wider range of diseases, including neurodegenerative conditions, metabolic syndromes, and cancer, where even small disruptions may increase the sensitivity of certain tissues. Equally crucial is uncovering how other organelles influence mitochondrial protein synthesis; emerging evidence suggests that ER-mitochondria contact sites, peroxisomal metabolism, and lysosomal signaling can tune mitochondrial translation through coordinated lipid exchange, calcium flux, and metabolite availability. Integrating these interactions with cellular stress pathways will be essential, as the ISR, ROS dynamics, and lipid homeostasis form an interconnected regulatory axis that modulates mitoribosome activity and translational fidelity under both physiological and stress conditions. A major unresolved question remains whether impaired mitochondrial translation is a primary causal factor in disease initiation or a downstream consequence of broader metabolic dysfunction; addressing this will require longitudinal models, patient-derived systems, and multi-omics analyses to distinguish early pathogenic triggers from compensatory responses.

Targeting mitochondrial translation regulation offers promising strategies for immune-related diseases. Mitochondrial replacement therapy (MRT) and mitochondrial transplantation and aim to restore translational capacity by implanting functional mitochondria with intact protein synthesis machinery, thereby rescuing OXPHOS, enhancing ATP production, and reducing oxidative stress. CRISPR-based editing, enabled by mitochondrial-import domains guiding RNA into mitochondria, now offers a means to correct mutated mtDNA directly. Advanced tools like mitoFUNCAT, mitoSunTag, and live-cell reporters now allow spatially resolved mapping of mitotranslational activity. Additionally, the small molecule PZL-A, currently in Phase I trials, has shown potential in restoring DNA polymerase-γ function and improving energy metabolism in POLG-related disorders. Emerging research on tRNA modifications further reveals how codon recognition and translation efficiency adapt during mitochondrial stress, offering novel therapeutic targets across inflammatory and mitochondrial diseases. Immunomodulation *via* agents such as ISRIB, mitochondrial-targeting antibiotics, or RNA modification inhibitors may help reprogram immune responses with precision. Future efforts must exploit lineage-specific mitochondrial translation features to achieve immune cell-selective modulation.

## Conflict of interest

The authors declare that they do not have any conflicts of interest with the content of this article.
